# Light-responsive paper strips as CO-releasing material with a colourimetric response†
†Electronic supplementary information (ESI) available. CCDC 1534335. For ESI and crystallographic data in CIF or other electronic format see DOI: 10.1039/c7sc01692a



**DOI:** 10.1039/c7sc01692a

**Published:** 2017-07-26

**Authors:** Upendar Reddy G., Jingjing Liu, Patrick Hoffmann, Johannes Steinmetzer, Helmar Görls, Stephan Kupfer, Sven H. C. Askes, Ute Neugebauer, Stefanie Gräfe, Alexander Schiller

**Affiliations:** a Institute for Inorganic and Analytical Chemistry (IAAC) , Friedrich Schiller University Jena , Humboldtstr. 8 , D-07743 Jena , Germany . Email: alexander.schiller@uni-jena.de; b Leibniz Institute of Photonic Technology , Albert-Einstein-Str. 9 , D-07745 Jena , Germany; c Center for Sepsis Control and Care (CSCC) , Jena University Hospital , Am Klinikum 1 , D-07747 Jena , Germany; d Institute of Physical Chemistry (IPC) , Abbe Center for Photonics Friedrich Schiller University Jena , Helmholtzweg 4 , D-07743 Jena , Germany

## Abstract

Carbon monoxide (CO) is known for its multifaceted role in human physiology, and molecules that release CO in a controlled way have been proposed as therapeutic drugs. In this work, a light-responsive CO-releasing molecule (**CORM-Dabsyl**) showed a strong colourimetric response upon photochemical CO-release, owing to the tight conjugation of a Mn(i) tricarbonyl centre to a dabsyl chromophoric ligand (**L**). Whereas the complex was very stable in the dark in nitrogen-purged aqueous media, CO-release was effectively triggered using 405 nm irradiation. **CORM-Dabsyl**, **L** and the inactive product **iCORM-Dabsyl** have been investigated by DFT and TD-DFT calculations. Only mild toxicity of **CORM-Dabsyl** was observed against LX-2 and HepaRG® human cell lines (IC_50_ ∼ 30 μM). Finally, to develop a CO storage and release material that is readily applicable to therapeutic situations, **CORM-Dabsyl** was loaded on low-cost and easily disposable paper strips, from which the light triggered CO-release was conveniently visible with the naked eye.

## Abstract 2

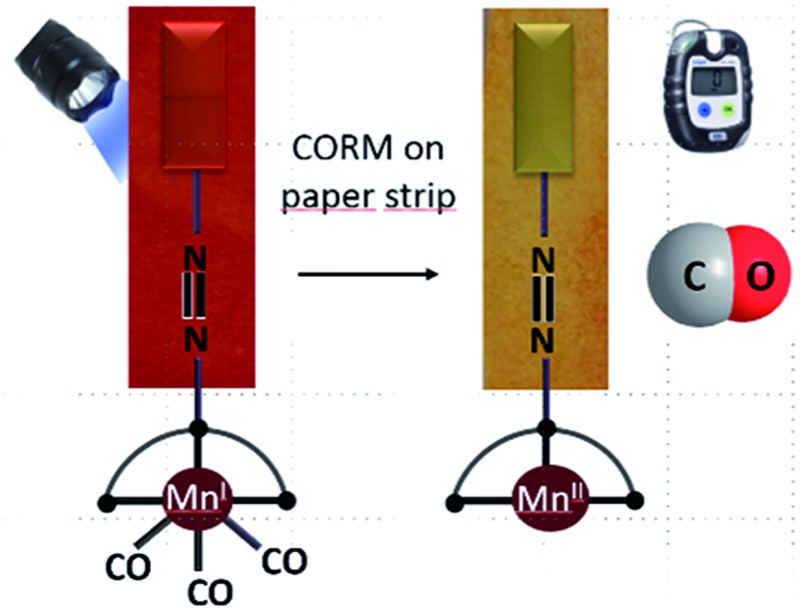
A light-responsive paper strip as CO-releasing material (CORMA) for convenient and efficient CO-release with colorimetric response.

## Introduction

In recent years, carbon monoxide (CO) has been identified as a vital messenger molecule in mammals, similar to nitric oxide (NO) and hydrogen sulfide (H_2_S), with physiological significance and potential as a therapeutic agent.^1^ CO imparts significant anti-inflammatory, cytoprotective and vasodilatory effects in mammalian physiology through various pathways.^2^ It also provides protection from myocardial infarction, has been employed to protect and preserve organs during transplantation procedures, and has been proposed as antibacterial and anticancer agent.^3^ Altogether, CO has been shown as an alternative drug to be employed when inflammation plays a damaging role. Despite the beneficial biological properties, the application of CO as a therapeutic agent is still in its infancy due to the technical challenge of delivering gaseous CO to a specific location with control over dosage and timing.^4^ Consequently, stable molecules that are able to store CO and release it upon an external stimulus (CO-releasing molecules; CORMs) are of great medical interest.

Several methodologies have been adopted for efficient CO-release which includes solvent-assisted CO-release,^5^ light-triggered CO-release (photo-CORMs),^6^ enzyme-triggered CO-release (ET-CORMs)^7^ and chemical-triggered CO-release (CT-CORM)^8^ that feature transition metal carbonyl complexes as possible prodrugs.^9,10^ However, localized administration of CO through a molecular CORM is difficult due to rapid diffusion, which may cause toxicity to untargeted healthy tissues; either because of released CO or metal and spectator ligand fragments that are released upon dissociation. These drawbacks motivated us and others to design hybrid polymer and inorganic matrices for CO-release (CO-releasing materials; CORMAs). The hybridization of carbonyl complexes into macromolecular or inorganic scaffolds may additionally stabilize the CORM or improve the cellular uptake.^11^ So far, photo-CORMs have been embedded in various macromolecular carriers such as dendritic structures,^12^ covalently immobilized onto the surfaces of nanoparticles^13^ and protein cages,^14^ or trapped in polymer fibers.^15^ Mascharak *et al.* recently incorporated photo-CORMs into pores of aluminosilicate nanoparticles.^16^ Ford and coworkers described water-soluble light-upconverting nanoparticles that were functionalized with a Mn(i) carbonyl complex, which released CO upon near-infrared irradiation.^17^ Recently, Kitagawa *et al.* reported a CO-releasing metal organic framework (CORF) based on a Mn(i) bipyridine tricarbonyl building block.^18^ Although these CORMAs are intriguing from a scientific point of view, they require elaborate and time-consuming fabrication methods and are therefore not readily applicable.

In addition to other carrier materials, paper strips are an alternative technology for fabricating simple, inexpensive, portable and disposable analytical devices, assembled from only filter paper and designed molecules.^19^ Due to the development of paper-based microfluidics,^19*b*^ paper strips have become a promising platform for lab-on-a-chip devices. These devices can act as portable diagnostic tools. Khan *et al.* described paper strips as low-cost material for blood sensors and blood group analysis,^19*c*^ Li *et al.* developed a paper-based testing disc for multiplex whole cell bacteria analysis.^19*d*^ For these reasons, we were interested in combining CORMs and paper strips for the development of a quickly and easily prepared CORMA in potential medical applications. To the best of our knowledge, release of CO from paper strips has not been reported yet.

In this work, a dabsyl moiety (*i.e.*, 4-(dimethylamino)azobenzene-4′-sulfonyl chloride) was tethered to di-(2-picolyl)amine (DPA) to synthesize a symmetrical tridentate ligand (**L**) that can coordinate facially to a Mn(i) carbonyl centre, thereby producing Mn(i) complex **CORM-Dabsyl**. As an overall strategy, dabsyl was selected as a strong chromophore, which originates from a strong intramolecular charge transfer in the excited state (tertiary amine to azobenzene-bridge),^20^ and conjugating it directly to the Mn(i)-centre to elicit a colour-change upon CO-release. Such a colourimetric response would allow for easy observation whether or not the molecule has released CO. In a next step, **CORM-Dabsyl** was immobilized on a paper strip to allow convenient and efficient CO-release upon blue light from the material.

## Results and discussion

**Fig. 1:**
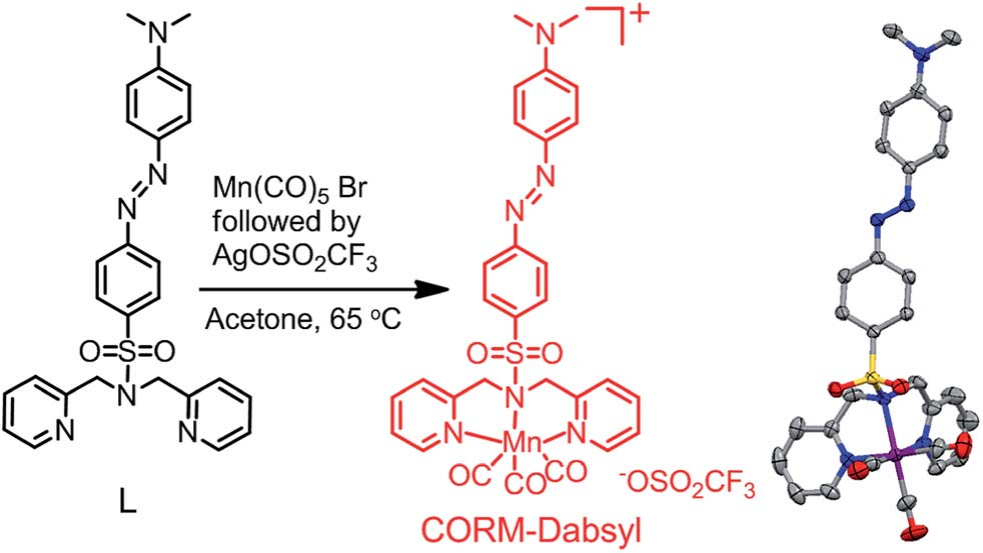
Reaction scheme for the synthesis of **CORM-Dabsyl**. X-ray crystal structure shown on the right. Solvent molecules, triflate anion and hydrogens in crystal structure are omitted for clarity; gray = carbon, blue = nitrogen, red = oxygen, purple = manganese and yellow = sulphur. For details, see the ESI.†

Analytical and spectroscopic data confirmed the successful synthesis and purity of ligand **L** and **CORM-Dabsyl** (ESI†). The solid-state X-ray crystal structure of **CORM-Dabsyl** shows that the three carbonyl ligands are facially coordinated to the Mn centre and opposite to the facially coordinated dipicolyl amine moiety (Fig. 1). The Mn–N(sp^3^) bond length (2.161(3) Å) was found to be unusually longer than the other Mn–N (sp^2^) bond distances (generally ∼2.050(4) Å to 2.052(4) Å) and confirms the superior coordinating ability of the sp^2^ hybridized pyridine nitrogens compared to the sp^3^ hybridized amine. The IR spectrum of **CORM-Dabsyl** displayed two *ν*_CO_ bands (2037 and 1928 cm^–1^) instead of three due to the high symmetry (no splitting at 1928 cm^–1^). Furthermore, comparison of ^1^H NMR spectra of **L** and **CORM-Dabsyl** shows that the methylene protons of the dipicolylamine moiety have singlet multiplicity for **L** and become two doublets for **CORM-Dabsyl**, which is explained by the fact that one pair projects towards and the other pair projects away from the carbonyl ligands.^8^ In addition, a complex with [Mn(CO)_3_]^+^ and dipicolyl(methyl)amine^8^ was used as control compound for unambiguous assignment of the spectral responses of **CORM-Dabsyl** upon release of CO molecules as well as the influence of the –SO_2_ unit and the dye moiety.

### UV-Vis absorption

**Fig. 2:**
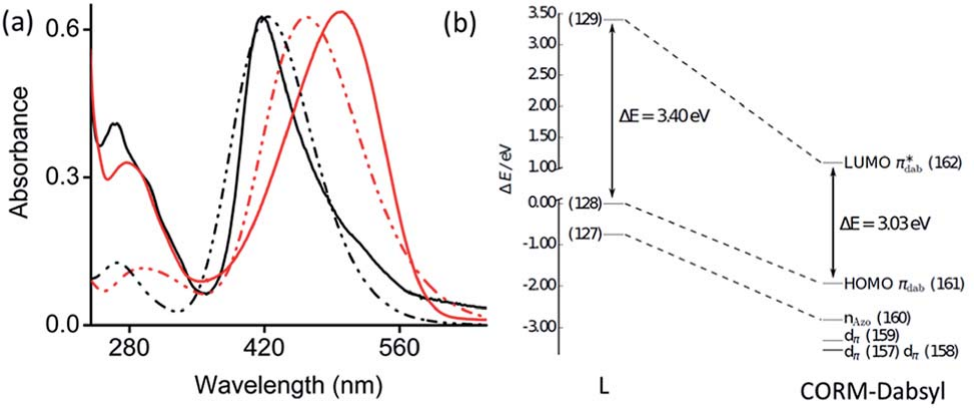
(a) Normalized experimental (solid) and theoretical (dashed) absorbance spectra of **L** (black) and **CORM-Dabsyl** (red) in aqueous phosphate buffer : DMSO (99 : 1 v/v, pH 7.4). (b) Energy scheme of the frontier orbitals of **L** and **CORM-Dabsyl** obtained at the M06/def2-TZVP level. All energies are in relation to the energy of the HOMO of **L** (MO 128). The orbital pairs (127, 160), (128, 161) and (129, 162) have the same character in **L** and **CORM-Dabsyl**. For details, see the ESI.†

The electronic absorption spectra of **L** and **CORM-Dabsyl** were measured in aqueous phosphate buffer PB : DMSO (99 : 1, v/v) solution and compared to calculated spectra obtained by the TD-DFT (Fig. 2a). Ligand **L** shows a strong absorption band at 415 nm (*ε* = 3.2 × 10^4^ mol^–1^ L cm^–1^), which is assigned to an intramolecular charge transfer (ICT) process from the *N*,*N*′-dimethylamine donor moiety to the azo (–N=N–) fragment by means of quantum chemical simulations. TD-DFT predicts a bright state (S_2_) at 424 nm that corresponds to the HOMO → LUMO ICT-transition on the dabsyl moiety. **CORM-Dabsyl** exhibits an intense absorption band in the visible region at 500 nm (*ε* = 3.3 × 10^4^ mol^–1^ L cm^–1^). TD-DFT estimates the bright excitation (S_0_–S_2_) at 464 nm, that corresponds to the same HOMO → LUMO transition, but with a distinct redshift compared to the ICT transition in **L** (Δ*E* = 0.37 eV, Δ*λ*_max_ = 84 nm).

When going from **L** to **CORM-Dabsyl**, coordination of the [Mn(CO)_3_]^+^ fragment leads to an introduction of a positive charge into the molecular system. Analyzing the atomic charges in **CORM-Dabsyl** using intrinsic atomic orbitals (IAO) reveals that the [Mn(CO)_3_] fragment possesses only a charge of +0.4*e* upon coordination.^21^ The majority of the positive charge (+0.6*e*) is delocalized over the coligand and leads to a stabilization of the frontier orbitals of **CORM-Dabsyl**. The HOMO of **CORM-Dabsyl** is stabilized by 1.94 eV, whereas the LUMO is stabilized by 2.31 eV, resulting in a smaller HOMO–LUMO gap in **CORM-Dabsyl** compared to **L** and explaining the observable redshift of the bright absorption feature assigned to the S_2_ state (Fig. 2b).

### Colourimetric response of **CORM-Dabsyl** and investigation of the CO-release mechanism

**Fig. 3:**
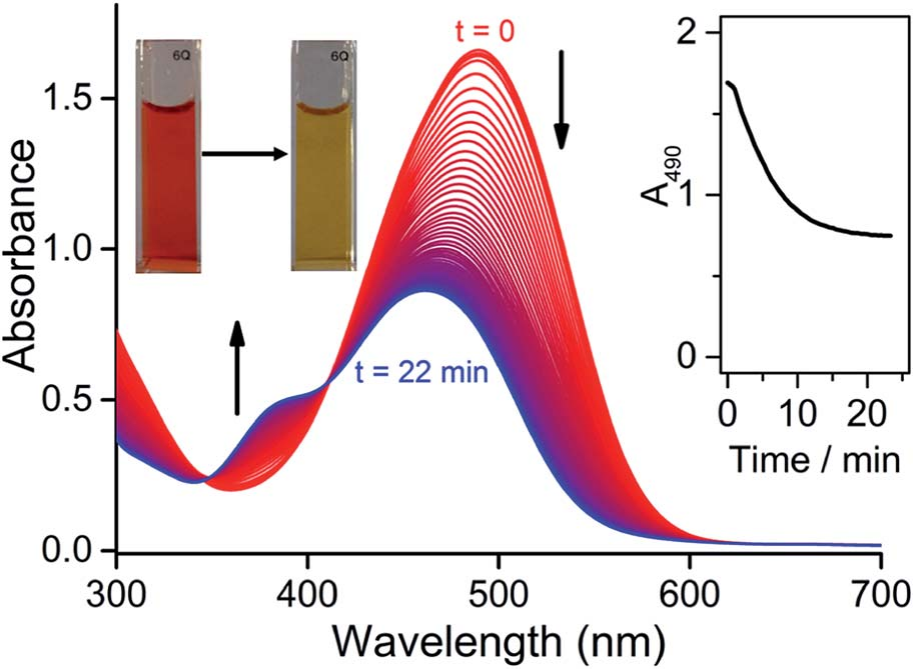
Evolution of absorption spectra of **CORM-Dabsyl** (50 μM) in DMSO solution during blue light irradiation (424 nm, 23 mW). Time between spectra is 40 s. Inset: absorbance at 490 nm as a function of irradiation time. Pictures show the solution in a cuvette before (left) and after (right) the photoreaction.

To investigate whether **CORM-Dabsyl** is able to photochemically release CO, a DMSO solution of **CORM-Dabsyl** (50 μM, 3 mL) was irradiated with 424 nm while recording UV-Vis absorption spectra every second. The absorption band at 490 nm was found to decrease gradually with a concomitant increase in absorbance around 385 nm. The colour of the solution changed from dark red to pale yellow (Fig. 3). A clear isosbestic point was observed at 411 nm, indicating that the photoreaction proceeds cleanly and gives only one photoproduct, without observable intermediate species. From these data, a photoreaction quantum yield of 0.5 ± 0.1% was calculated (Experimental section). The photoreaction was also performed in phosphate buffer (99 : 1 PB : DMSO, Fig S10a†). The same reduction in 490 nm absorbance was observed with at a very similar initial reaction rate, but the photoreaction could not be studied in detail due to precipitation of the photoproduct in this medium. Due to the similar reaction rate, we assume that the photoreaction quantum yield is similar in phosphate buffer and in neat DMSO. Meanwhile, in dark conditions in phosphate buffer no changes in absorbance were observed over a course of 12 h (Fig. S10†), showing an exceptional stability of **CORM-Dabsyl** *versus* oxidation and decomposition in solution. Moreover, no spectral changes were observed when only ligand **L** was irradiated at 405 nm (Fig. S11†), which mitigates the possibility that photo-induced *trans*–*cis* isomerization plays a role in the observed spectral changes during irradiation of **CORM-Dabsyl**.

**Fig. 4:**
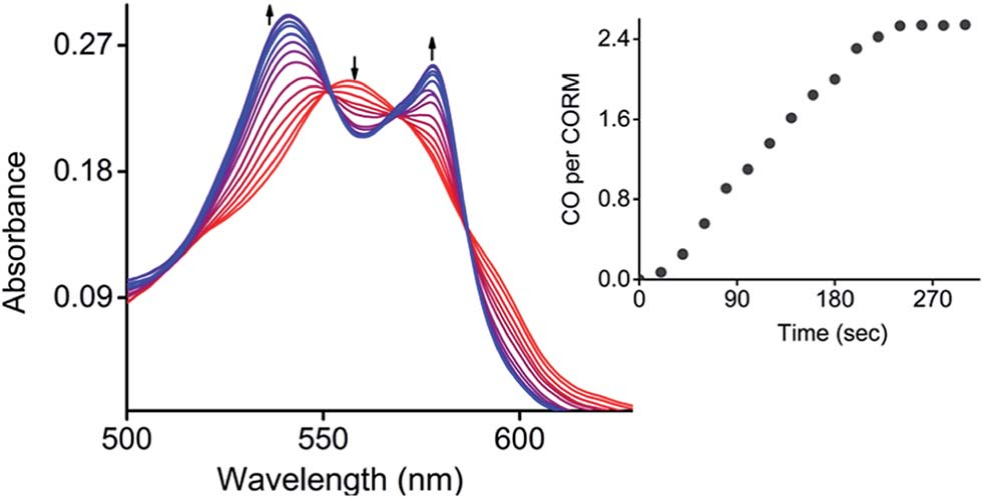
Conversion of deoxy-Mb to Mb–CO in a mixture of **CORM-Dabsyl** (15 μM) and deoxy-Mb (60 μM) in phosphate buffer (pH 7.4) upon exposure to light (405 nm, 8 mW cm^–2^). Inset: CO release per **CORM-Dabsyl** *versus* time.

Next, the photo-induced release of CO was investigated using a myoglobin (Mb) assay based on the spectral changes in absorption when deoxy-Mb is converted to Mb–CO (Fig. 4).^15^ Briefly, a freshly prepared concentrated solution of **CORM-Dabsyl** in DMSO was added to a buffered solution of horse skeletal myoglobin (Mb), which had been reduced with sodium dithionite in absence of oxygen. Upon illumination of this mixture with light (405 nm, 8 mW cm^–2^), the absorbance of deoxy-Mb at 557 nm decreased and the characteristic absorbance bands of Mb–CO at 540 and 577 nm grew and stabilized after 4 min, thereby confirming the binding of CO to Mb. To know the influence of sodium dithionite on CO-release from **CORM-Dabsyl**, a reference myoglobin assay setup was kept in the dark for 60 min, where no absorbance changes at 540 and 577 nm were observed (Fig. S12†). This confirms not only the stability of **CORM-Dabsyl** in the dark, but also the stability against sodium dithionite. Hence, the release of CO was actually induced by light illumination and not by the sodium dithionite. Thus, these data demonstrate that **CORM-Dabsyl** successfully releases CO upon irradiation. The amount of CO per CORM was calculated from these data and amounted to the near-complete release of three equivalents CO for each molecule **CORM-Dabsyl** (Fig. 4). The previously calculated photoreaction quantum yield was therefore multiplied by 3, which gave a quantum yield of CO-release of 1.5 ± 0.2% (with 424 nm irradiation).

To identify which molecular orbitals are associated to the photochemical release of CO upon blue light illumination, TD-DFT calculations were performed. Several excited states (S_4_–S_6_) were identified between 418 and 396 nm that correspond to excitations from the three (Mn–CO)-bonding d_π_-orbitals (MOs 157–159), responsible for π-backbonding to CO, to (Mn–CO)-antibonding orbitals (MOs 167 and 168, Fig. S20†). While the d_π_-orbitals feature mainly d-character, the MOs 167 and 168 correspond to antibonding linear combinations between 
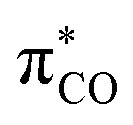
- and the e_g_-orbitals.^22^ These data suggest that the photochemical activity of **CORM-Dabsyl** originates from orbitals around the Mn centre and is not occurring from ligand excited states that are populated with light above 418 nm (up to 600 nm, recall Fig. 2). Indeed, upon excitation with 480 nm light, which is very strongly absorbed by the dabsyl moiety in **CORM-Dabsyl**, no CO-release is observed at all (Fig. S13b†). This result clarifies that the complex only reacts with deep-blue light and that the dabsyl functions act as colourimetric marker without contributing to the photochemical release of CO, however, the charge distribution in vicinity of the Mn centre (electronic ground state) is highly influenced by means of the CT character of **L**. Overall, it was established that **CORM-Dabsyl** is a highly stable CORM that releases all three carbonyls upon low intensity light exposure, while a marked colourimetric response can be easily observed by the naked eye.

### Characterization of the inactive product after irradiation

The photochemical reaction of **CORM-Dabsyl** produces not only CO, but also an “inactive” Mn product (iCORM), which was characterized by ESR, NMR, IR spectroscopy, as well as by ESI-MS (ESI†) and computational methods. The X-band ESR spectrum (at 295 K) of the photolyzed solution of **CORM-Dabsyl** exhibits a six-line spectrum indicative of a paramagnetic Mn(ii) species (d^5^, high spin, sextet, Fig. S14†). The paramagnetic nature of the product was also confirmed by ^1^H NMR, which showed that all proton signals of **CORM-Dabsyl** broadened upon irradiation. However, no shifts of the proton signals were observed, which confirmed that ligand **L** is still bound to the Mn centre. In addition, ^13^C NMR spectroscopy revealed that the signal for the CO molecules of **CORM-Dabsyl** at 217.8 ppm disappeared upon irradiation of **CORM-Dabsyl** (Fig. S15†). Next, a solution of **CORM-Dabsyl** in dichloromethane was photolyzed with blue light (405 nm, 8 mW cm^–2^) and the dried residue was examined with IR spectroscopy. No CO stretches were observed in the entire 1900–2100 cm^–1^ region, which clearly confirms that all three CO ligands were released from **CORM-Dabsyl** (Fig. S16†). This is in accordance with data from the Mb-assay in aqueous solution (see Fig. 4). However, HRMS reported that the iCORM, generated by irradiation of solid **CORM-Dabsyl**, had a mass of 541.1222 *m*/*z*. This value was attributed to [**L** + Mn] with a Mn(i) species (Fig. S17†). Overall, these data show that upon irradiation and CO-release, the Mn centre is still bound to the ligand. The oxidation state I or II in the iCORM is dependent on the reaction conditions. We speculate that the three CO ligands are replaced by three H_2_O ligands in aqueous media, leading to **iCORM-Dabsyl**, *i.e.* [Mn**L**(H_2_O)_3_]^2+^. Unfortunately, we were not able to isolate or synthesize the inactive product as Mn(ii) complex. However, the different possible spin states of **iCORM-Dabsyl** were investigated by DFT calculation. Considering the first three doublet, quartet and sextet states, within their equilibrium structures, MS-RASPT2 predicts a high spin sextet ground state, energetically favoured with respect to the doublet (+1.79 eV) and quartet (+2.48 eV) ground state (ESI Table 3†). In agreement with ESR, the sextet ground state corresponds to a 3d^5^ configuration on the Mn. Subsequently, the nature of the bright excitations underlying the absorption spectrum of the photoproduct were unraveled by means TD-DFT within sextet multiplicity. For **iCORM-Dabsyl** TD-DFT estimates two bright states (S_9_ and S_11_) at 496 nm and 477 nm. Their character is similar to the bright S_2_ state as in **L** and **CORM-Dabysl** (ESI, Tables 1 and 2†) with additional charge-transfer from the azobenzene into the 
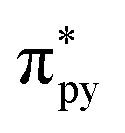
-orbitals.

### Toxicity of **CORM-Dabsyl** against human cell lines

**Fig. 5:**
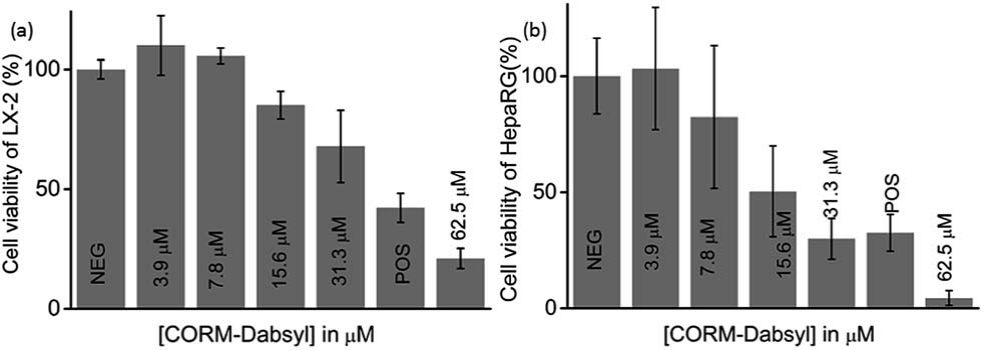
Relative percentage of cell viability of (a) LX-2 cells and (b) HepaRG® cells that were treated for 24 h with medium only (NEG), 5 μM alpha-toxin (POS), or a concentration series of **CORM-Dabsyl** (3.9 μM to 62.5 μM).

To determine the potential cytotoxicity, **CORM-Dabsyl** was tested against two human hepatic model cell lines (HepaRG® and LX-2) by applying a common resazurin-based cell viability assay in the dark at standard cell culture conditions (Fig. 5).^8^ *In vitro* experiments revealed that **CORM-Dabsyl** is mildly cytotoxic to both cell lines with EC_50_ values of 31 ± 5 μM (LX-2) and 26 ± 7 μM (HepaRG®) for an incubation time of 24 h. Nevertheless, these results convey the need for CORMs to be immobilized in a carrier material in order to prevent or reduce cytotoxic effects at the desired site of CO-release.

### Light-induced CO-release from paper strips

**Fig. 6:**
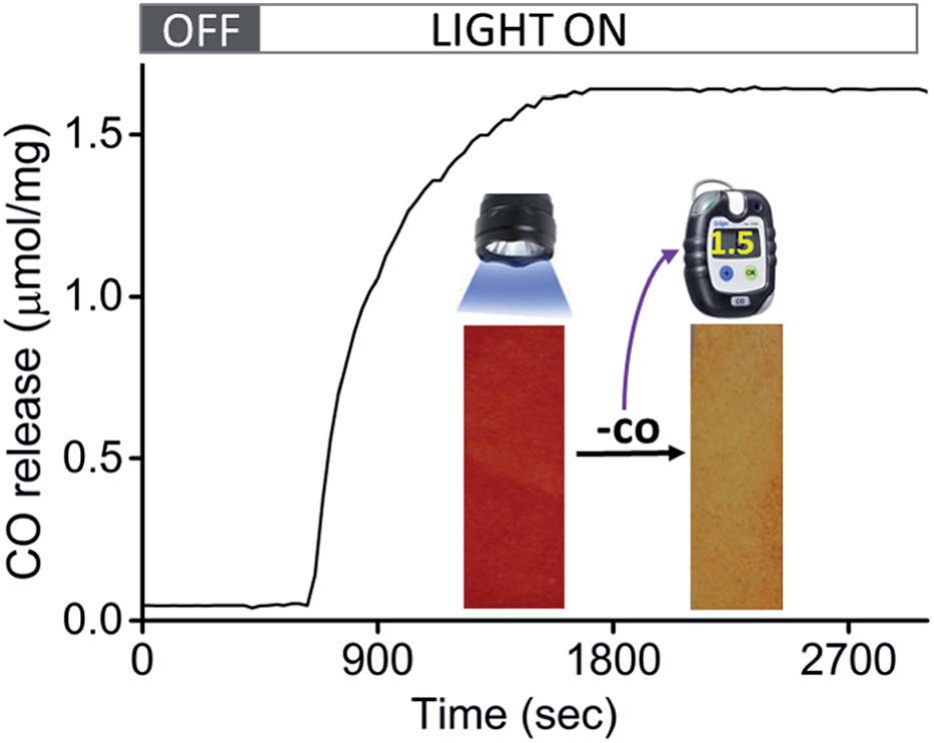
Amount of CO released as a function of time during irradiation with LED light (*λ*_irr_ = 405 nm; 8 mW cm^–2^) of paper strips loaded with **CORM-Dabsyl** in a closed desiccator. The inset shows the colour change from red (before) to yellow (after) due to irradiation.

Encouraged by the efficacious photoreactivity and stability of **CORM-Dabsyl**, we endeavoured to develop a low-cost and photoactive CO delivery material with a colourimetric feedback. A 1 × 3 cm paper strip was soaked in a DCM solution (0.5 mL) of **CORM-Dabsyl** (0.5 mg). Within 2 min the entire fluid had been homogeneously absorbed due to capillary forces. The red paper was air-dried for 3–4 min. The colour of the paper strips remained unchanged during several weeks in the open air, which indicates the stability of **CORM-Dabsyl** in the material. CORM leaching in aqueous solution within 15 min was negligible (Fig. S19†). In order to investigate whether CO could be photochemically produced from the paper strips, the strips were irradiated in a closed desiccator with 405 nm LED-lights (8 mW cm^–2^), while monitoring the CO concentration with a portable CO-sensor.^15^ Interestingly, upon exposure of light (*λ*_irr_ = 405 nm; 8 mW cm^–2^) the strips turned from red to yellow (Fig. 6), similar to the colour changes observed previously for **CORM-Dabsyl** in solution. The amount of CO-release from one paper strip (0.5 mg of **CORM-Dabsyl** loaded) under illumination within 10 minutes, was 1.5 μmol mg^–1^.

To furthermore confirm the CO-release, the paper strip was characterized with ATR-IR spectroscopy immediately after light exposure (Fig. S18†). The CO vibration bands that were present before irradiation (1900–2100 cm^–1^) had completely vanished as a result of light exposure. As a control, the experiment was repeated in ambient light conditions without LED irradiation: no released CO was detected during 16 h measurement time (Fig. S18†). Overall, these results signify that CO is only released upon photolysis (*λ*_irr_ = 405 nm; 8 mW cm^–2^). The strategy of using a paper strip with an immobilized photo-CORM is highly promising for the development of a simple, inexpensive, and portable CO-releasing wound cover that may be used to release CO on demand at a disease site. Moreover, the clearly visible colourimetric response of the paper strips give instant feedback whether or not the CO has been released.

## Conclusions

In brief, we have reported a stable and water-soluble colourimetric CO-releasing molecule (**CORM-Dabsyl**) that releases three CO molecules upon photolysis. The associated colour change is easily observed by the naked eye. DTF and TD-DFT calculations gave insight into the CO release mechanism, dye contribution and the properties of the inactive CORM. For the first time, paper strips based on **CORM-Dabsyl** were fabricated, which could act as a CO-releasing material for convenient and efficient CO-release under light illumination with a strong colourimetric response. These paper strips are highly useful for on-demand release of CO at a biological site, while it is directly visible whether CO has been released or not. The ease of preparation, the cost-effectiveness, and the stability of the material render the paper strips immediately applicable to medical situations where CO is needed as drug, and will pave the way for a next generation of CO-releasing materials.

## Conflict of interest

There are no conflicts of interest to declare.
