# Supplementary material for: Light-responsive paper strips as CO-releasing material with a colourimetric response
Source: Chem Sci. 2017 Jul 26;8(9):6555–60. doi: 10.1039/c7sc01692a (PMC5627354; doi:10.1039/c7sc01692a)
Supplement: Supplementary file 1 [file SC-008-C7SC01692A-s001.pdf]

Electronic Supplementary Material (ESI) for Chemical Science.
This journal is © The Royal Society of Chemistry 2017

Electronic Supporting Information

Light-responsive paper strips as CO-releasing material with a colorimetric response

Upendar Reddy G[a], Jingjing Liu[a], Patrick Hoffmann[b,c], Johannes Steinmetzer[d], Helmar Görls[a], Stephan Kupfer[d], Sven H. C. Askes,[a] Ute Neugebauer[b,c], Stefanie Gräfe[d], Alexander Schiller*[a]

[a]Institute for Inorganic and Analytical Chemistry (IAAC), Friedrich Schiller University Jena, Humboldtstr. 8, D-07743 Jena, Germany. E-mail: alexander.schiller@uni-jena.de

[b]Leibniz Institute of Photonic Technology, Albert-Einstein-Straße 9, D-07745 Jena, Germany.

[c]Center for Sepsis Control and Care (CSCC), Jena University Hospital, Am Klinikum 1, D-07747 Jena, Germany

[d]Institute of Physical Chemistry (IPC) and Abbe Center for Photonics, Friedrich Schiller University Jena, Helmholtzweg 4, D-07743 Jena, Germany.

Table of contents:	Page
Experimental details	S3
Synthetic scheme	S8
^{1}H NMR spectrum of **L**	S8
^{13}C NMR spectrum of **L**	S9
ESI-MS spectrum of **L** and IR spectrum of **L**	S10
^{1}H NMR spectrum of **CORM-Dabsyl**	S11
^{13}C NMR spectrum of **CORM-Dabsyl**	S12
ESI-MS spectrum and ^{19}F NMR spectrum of **CORM-Dabsyl**	S13
IR spectrum of **CORM-Dabsyl** and UV-Vis spectral changes of **CORM-Dabsyl** at photolysis as well as dark conditions	S14
UV-Vis spectral changes of **L** upon illumination at 405 nm and influence of sodium dithionite for **CORM-Dabsyl** on CO-releasing	S15
UV-Vis spectral changes of **CORM-Dabsyl** at different irradiation wavelengths and ESR spectrum of **CORM-Dabsyl** before and after irradiation	S16
^{1}H NMR and ^{13}C NMR spectral changes of **CORM-Dabsyl** before and after irradiation and IR spectrum of **CORM-Dabsyl** before and after irradiation	S17
ESI-MS spectra of **CORM-Dabsyl** after irradiation and stability of paper strip and IR response of paper strip before and after irradiation	S18
CO leackage experiment from paper strip<br>Theoretical Results	S19
Crystal structure of **CORM-Dabsyl**	S26
References	S26

Experimental details

Materials and instrumentation. $Mn(CO)_5Br$, silver triflate, di-(2-picolyl)amine, dabsyl chloride and triethylamine were purchased from Sigma-Aldrich. Solvents were purified and/or dried by standard techniques prior to use for all syntheses. ^1H NMR, ^{13}C NMR and ^{19}F NMR spectra were recorded on a Bruker AV 400 MHz NMR spectrometer in $CDCl_3$ or DMSO-d_6. Tetramethylsilane (TMS) and trichlorofluoromethane (CCl_3F) were used as internal standards for ^1H NMR and ^{19}F NMR, respectively. UV-Vis absorption spectra were recorded on an Analytik Jena Specord S 600 UV-Vis spectrometer. Mass spectra were acquired on a Finnigan MAZ95XL device. Elemental analysis was performed on a Vario EL III CHNS instrument. All IR (ATR) spectra were recorded on a Bruker Vertex 70 FT-IR spectrometer. Electron spin resonance (ESR) spectra on solution samples were recorded at X-band frequencies on a Bruker Elexsys E500 spectrometer. High resolution mass spectrometry was carried out using a Thermo (Bremen, Germany) QExactive plus Orbitrap mass spectrometer coupled to a heated electrospray source (HESI). Flow was set to 25 µL/min. For monitoring two full scan modes were selected with the following parameters. Polarity: positive; scan range: 100 to 1500 *m*/*z*; resolution: 280,000; AGC target: 3×10^6; maximum IT: 200 ms.

General experimental methods for UV-Vis studies. The 1 mM stock solutions of **CORM-Dabsyl** and **L** were prepared in DMSO, which was used for all studies. For spectroscopic measurements, the DMSO stock solution was diluted with 10 mM phosphate buffer (PB, pH 7.4) to acquire a 15 µM solution in 1:99 *v/v* DMSO:PB, unless not otherwise mentioned. UV-Vis absorption spectra were recorded at room temperature in a quartz cuvette every 20 sec during UV light exposure (UVP Benchtop 2 UV Transilluminator, ≤ 8 mW cm^{-2}).

Photolysis experiment. Solutions of **CORM-Dabsyl** (15 µM) in a quartz cuvette were exposed to different light sources (365 nm, 405 nm and 480 nm; 8 mW cm^{-2}) at every 20 sec intervals to investigate the photoinduced CO-release. The power of the light sources was determined with a PM100USB power meter.

CO-release study. The light induced CO-release from paper strips was achieved by illumination with LED light (405 nm) in closed desicator.[1a] The irradiation experiments were performed at fixed light intensities (8 mW cm^{-2}). The light induced CO-release could detected by a PAC7000 electrochemical CO Sensor (Drägerwerk AG & Co. KGaA, Lübeck, Germany). The amount of detected CO from triplicate measurements was re-calculated using the ideal gas equation and expressed as µmol CO per mg sample.[1b] The number of equivalents CO-release per CORM-Dabsyl was determined by myglobin experiment by using the following equation.[1c]

$$c(\text{MbCO}) = \left\{\frac{A(t)}{l} - \frac{A(t=0)}{l}\right\} \frac{1}{\varepsilon_{540nm}(MbCO) - \dfrac{\Delta(t=0)}{c_0(Mb)\cdot l}}$$

Where t is the time, *l* is the path length of the cuvette, c_0(Mb) is the initial concentration of myoglobin ($\varepsilon_{540\ nm}$ = 15.4 $mM^{-1}cm^{-1}$).[1c] The calculated MbCO concentration was plotted against the illumination

time. The calculated MbCO concentration devided by the **CORM-Dabsyl** concentration to get the number of CO equivalents released per mole of **CORM-Dabsyl**.[1c]

Cytotoxicity measurements. Cytotoxicity of **CORM-Dabsyl** was quantified using a cell metabolic activity assay. Two well-established human cell lines were used: hepatic stellate cell-line LX-2 and hepatic stem cell line (differentiated) HepaRG®. LX-2 cells were cultivated in DMEM supplemented with fetal calf serum (10%, *v*/*v*), penicillin (100 U/mL), and streptomycin (100 µg/mL). HepaRG® cells were differentiated and cultured in Williams' medium E supplemented with fetal calf serum (10%, *v*/*v*), glutamine (2 mM), hydrocortisone 21-hemisuccinate (50 µM), DMSO (2%, *v*/*v*), human recombinant insulin (10 µg/mL), penicillin (100 U/mL), and streptomycin (100 µg/mL). Standard cell culture conditions of 37.0 °C, 5.0% CO_2, and a humidified atmosphere were used. Differentiated HepaRG® cells were plated in black 96-well plates with a density of 80,000 cells/well and for LX-2 cells 20,000 cells/well were seeded. Subsequently, the cells were allowed to adhere for 24 h. Then, the medium was exchanged with 100 µL of **CORM-Dabsyl** (3.9 to 62.5 µM) in 1:99 *v/v* DMSO:cell medium. *Alpha*-toxin (5 µg/mL, Sigma-Aldrich) was applied as positive control (POS) to provide a reproducible cytotoxic response, and cell culture medium was selected as negative control (NEG). After 24 h incubation, 10 µL AlamarBlue (Life Technologies) were added to each well and the 96-well plates were incubated for 1 h, after which the cell viability was assessed by measuring the fluorescence intensity at 590 nm (λ_{ex} = 560 nm) using an Infinite 200 Pro microplate reader (Tecan). The experiments were carried out in quadruplicates and all experimental steps were executed under exclusion of direct light. The fluorescence raw data sets were analyzed by the Grubbs test and outliers detected for a confidence interval of 95% were removed. The recorded fluorescence signal was directly proportional to the metabolic activity of the cells and was normalized to the negative control to obtain the viability in each well. Additionally, raw values of the diluted **CORM-Dabsyl** solutions were subtracted for each sample to avoid false positive results by potential fluorescence or absorbance by **CORM-Dabsyl**. The EC_{50} value for each cell line, *i.e.*, the concentration at which 50 % viability was observed, was computed by fitting a predefined sigmoidal log-dose response curve in Origin (OriginLab, Northampton, MA).

Synthetic procedures

Synthesis of (E)-4-((4-(dimethylamino)phenyl)diazenyl)-*N,N*-bis(pyridin-2-ylmethyl)benzene-sulfonamide (L). Di-(2-picolyl) amine (105 mg, 0.5 mmol) and dabsyl chloride (162 mg, 0.5 mmol) were dissolved in 25 mL of dry tetrahydrofuran. To this solution, triethylamine (0.1 mL) was added and allowed to stir for 5 h at room temperature. A red-coloured precipitate was filtered. The filtrate was concentrated and subjected to silica column chromatographic (ethyl acetate:*n*-hexane in 1:1 *v*/*v*) purification to yield pure **L** as a red solid (150 mg, 61%). HRMS calculated for $C_{26}H_{26}N_6O_2S$ [L+H$^+$]: 487.1911, observed: 487.1918. ^{1}H NMR [400 MHz, $CDCl_3$: δ (ppm)]: 8.39 (2H, d, *J* = 4.4 Hz, ArH); 7.94-7.87 (6H, m, ArH); 7.57 (2H, t, *J* = 7.2 Hz, ArH); 7.35 (2H, d, *J* = 7.6Hz, ArH); 7.10 (2H, t, *J* =

5.2 Hz, ArH); 6.79 (2H, d, J = 9.2Hz, ArH); 4.66 (4H, s, CH_2); 3.15 (6H, s, CH_3). ^{13}C NMR [100 MHz, $CDCl_3$]: δ (ppm) 156, 155, 149, 143, 139, 136, 128, 125, 122, 122, 122, 111, 53 and 46 (assignment see Fig. S1 and S2). Elemental analysis calculated for $C_{26}H_{26}N_6O_2S$: C = 64.18 %, H = 5.39 %, N = 17.27 %, S = 6.59 %, found: C = 62.11 %, H = 6.03 %, N = 13.62 %, S = 5.11 %. Contamination with salts and solvents possible.

Synthesis of [MnL(CO)$_3$](CF_3SO_3); CORM-Dabsyl. In the dark and under a nitrogen atmosphere, $Mn(CO)_5Br$ (110 mg, 0.40 mmol) and silver triflate (120 mg, 0.40 mmol) were dissolved in 15 mL dry acetone. The reaction mixture was stirred at 60 °C for 1.5 h. The reaction mixture was filtered under an inert atmosphere to separate the precipitate of silver bromide. To the filtrate, ligand **L** (150 mg, 0.31 mmol) in 10 mL of dry and deoxygenated acetone was added and the reaction mixture was heated under reflux for 60 °C for 1.5 h. The reaction mixture was filtered and the residue was washed with dry diethyl ether for several times to get pure **CORM-Dabsyl** as a dark red solid (180 mg, 74%). Slow diffusion of ether into a $CHCl_3$ solution of **CORM-Dabsyl** afforded orange red plate-like crystals suitable for X-ray crystallography. HRMS calculated for $C_{29}H_{26}MnN_6O_5S$ [(**CORM-Dabsyl**)-$^{-}OSO_2CF_3$]: 625.1060, observed: 625.1063. ^1H NMR [400 MHz, DMSO-d$_6$: δ (ppm)]: 8.95 (1H, d, J = 8.0 Hz, ArH); 8.84 (2H, d, J = 5.6 Hz, ArH); 8.62 (1H, t, J = 8.8 Hz, ArH); 8.46 (1H, d, J = 7.6 Hz, ArH); 7.44-7.78 (3H, m, ArH); 7.67 (1H, t, J = 8.0 Hz, ArH); 7.48 (2H, d, J = 7.6 Hz, ArH); 7.37 (2H, t, J = 6.0 Hz, ArH); 7.27 (1H, d, J = 8.8 Hz, ArH); 5.22 (2H, d, J = 16.4 Hz, CH_2); 4.35 (2H, d, J = 16.4 Hz, CH_2); 3.00 (6H, s, CH_3). ^{13}C NMR [125 MHz, DMSO-d$_6$]: δ (ppm) 217, 159, 157, 154, 143, 140, 133, 127, 126, 126, 124, 123, 112, 63 and 29 (assignment see Fig. S5 and S6). $^{19}F\{^1H\}$NMR (188 MHz, DMSO-d$_6$) δ-77.89; IR (ATR) ν = 2037 cm^{-1} and 1928 cm^{-1}. Elemental analysis calculated for $C_{30}H_{26}F_3MnN_6O_8S_2$: C = 46.52 %, H = 3.38 %, N = 10.85 %, S = 8.28%, found: C = 46.07 %, H = 3.39 %, N = 10.50 %, S = 8.03 %.

X-ray crystallography data. The intensity data were collected on a Nonius KappaCCD diffractometer, using graphite-monochromated Mo-K_α radiation. Data were corrected for Lorentz and polarization effects; absorption was taken into account on a semi-empirical basis using multiple-scans.[2] The structure was solved by direct methods (SHELXS) and refined by full-matrix least squares techniques against Fo^2 (SHELXL-2016).[3] All hydrogen atoms were included at calculated positions with fixed thermal parameters. All non-disordered, non-hydrogen atoms were refined anisotropically.[4] The crystal of **CORM-Dabsyl** contains large voids, filled with disordered solvent molecules. The size of the voids are 144 Å^3/unit cell. Their contribution to the structure factors was secured by back-Fourier transformation using the SQUEEZE routine of the program PLATON.[5] resulting in 39 electrons/unit cell. Mercury was used for structure respresentation.[6]

Crystal Data for CORM-Dabsyl. $C_{31}H_{30}F_3MnN_6O_9S_2$[*], Mr = 806.67 g mol^{-1}[*], orange prism, size 0.134 x 0.122 x 0.108 mm^3, triclinic, space group P $\bar{1}$, a = 10.5628(2), b = 13.4613(3), c = 26.2818(5) Å, α = 79.225(1), β = 83.349(1), γ = 75.404(1)°, V = 3543.33(12) Å^3 , T= -140 °C, Z = 4, $\rho_{calcd.}$ = 1.512

g cm^{-3}[*], μ (Mo-K$_{\alpha}$) = 5.67 cm^{-1}[*], multi-scan, transmin: 0.6886, transmax: 0.7456, F(000) = 1656[*], 26160 reflections in h(-13/13), k(-17/17), l(-33/33), measured in the range 1.58° ≤ Θ ≤ 27.10°, completeness Θmax = 95.9%, 15002 independent reflections, R_{int} = 0.0266, 12901 reflections with $F_o > 4\sigma(F_o)$, 925 parameters, 0 restraints, $R1_{obs}$ = 0.0867, wR^2_{obs} = 0.2193, $R1_{all}$ = 0.0985, wR^2_{all} = 0.2279, GOOF = 1.041, largest difference peak and hole: 2.815 / -3.716 e Å^{-3}. [*] derived parameters do not contain the contribution of the disordered solvent.

Supporting Information Available: Crystallographic data deposited at the Cambridge Crystallographic Data Centre under CCDC-1534335 for **CORM-Dabsyl** contain the supplementary crystallographic data excluding structure factors; this data can be obtained free of charge via www.ccdc.cam.ac.uk/conts/retrieving.html (or from the Cambridge Crystallographic Data Centre, 12, Union Road, Cambridge CB2 1EZ, UK; fax: (+44) 1223-336-033; or deposit@ccdc.cam.ac.uk).

Computational studies. All calculations performed at the density functional (DFT) and time-dependent density functional level of theory TD-DFT were carried out using TURBOMOLE 7.1.[7] Geometries, vibrational frequencies and normal coordinates of **L**, **CORM-Dabsyl** and a possible photoproduct ([Mn**L**$(H_2O)_3]^{2+}$, **iCORM-Dabsyl**), were obtained at the TPSS/def2-TZVP level of theory.[8,9] To speed up the calculations the RIJ and MARIJ approximations were employed.[10] Dispersion correction was included using the D3-method with Becke-Johnson damping.[11] The effect of a solvent (water, ε = 80.4, n = 1.33) on the molecular geometries and vibrational spectra was taken into account by the COSMO model.[27] Excited state properties (vertical excitation energies, oscillator strengths, leading electronic transitions) were obtained from TD-DFT calculations at the M06/def2-TZVP level of theory using the TPSS-geometries, while the RIK approximation was utilized for all M06-calculations.[12] For **L, CORM-Dabsyl** and **iCORM-Dabsyl** the lowest 80 electronically excited states were calculated in their respective multiplicity. Singlet multiplicity was assumed for all calculations on **L** and **CORM-Dabsyl**. Sextet multiplicity was assumed for **iCORM-Dabsyl** (see below). The calculated excitations were broadened with Gaussians with a full width at half maximum of 0.4 eV. The possible photoproduct (**iCORM-Dabsyl**) was also investigated using multiconfigurational methods, namely utilizing the SA-RASSCF (State average restricted active space self-consistent field)[13] and the MS-RASPT2 (second-order multi-state perturbational theory on a RASSCF reference wavefunction)[14] methodologies as implemented in Molcas 8.0,[15] on the fully optimised DFT geometry. Scalar relativistic effects were taken into account by a second-order Douglas-Kroll Hamiltonian.[16] The ANO-RCC-VDZP basis was used for all atoms but manganese, for which the corresponding triple-ζ basis was used.[17] To speed up the calculations the two-electron integrals were treated using the atomic compact Cholesky decomposition.[18] In order to avoid intruder states a real level shift of 0.3 a.u. was applied in all MS-RASPT2 calculations. To specify the restricted active space the RAS($n,l,m;i,j,k$) notation of Gagliardi and co-workers is employed.[19] Index n specifies the total number of active electrons in all subspaces.

Indices *l* and *m* give the maximum number of holes in RAS1 and the maximum number of electrons in RAS3, respectively. The number of molecular orbitals in the respective subspaces (RAS1, RAS2 and RAS3) is indicated by the indices *i*, *j* and *k*. For **iCORM-Dabsyl** a RAS(15,2,2;4,7,3) partitioning of the active space was used (Supporting Information). RAS2 contains the five manganese 3d orbitals and a pair of correlating $\pi_{dab}\pi_{dab}^*$ orbitals. RAS1 consists of a nonbonding orbital centered on the azo-nitrogens (n_{Azo}) and three π_{dab} orbitals. The corresponding set of three correlating π_{dab}^* orbitals is assigned to RAS3. For the state-average procedure the first three electronic states of **iCORM-Dabsyl** were considered.

Calculation of the quantum yield of photoactivation. A solution of **CORM-Dabsyl** in DMSO (50 µM, volume V = 3.0 mL) was transferred to a stirred quartz fluorescence cuvette (1 cm pathlength) at 20 °C in a UV-Vis absorption spectrometry setup. Irradiation was performed with a high power 424 nm fiber-coupled LED (Thorlabs M420F2) that was connected to a collimating lens on top of the cuvette, thereby producing a 23.3 mW light beam through the solution (i.e. photon flux $\Phi = 8.26 \times 10^{-8}$ einstein/s). UV-Vis spectra were recorded every second until the reaction was completed within 25 min. The quantum yield (φ) of the photochemical reaction of **CORM-Dabsyl** was then calculated according to Equation 1.[20] It was assumed that the photoreaction proceeds with pseudo-first order kinetics, which is justified by the fact that we observe the spectral evolution of one species to the other without any intermediates.

$$\varphi = \frac{k * n_{tot}}{\Phi * (1 - 10^{A_{424}})} \; (Equation\ 1)$$

where k is the reaction rate constant, n_{tot} is the total amount of manganese complex in solution, and A_{424} is the absorbance at 424 nm. k was obtained from the slope of a plot of ln([**CORM-Dabsyl**]/(n_{tot}/V)) versus irradiation time, where [**CORM-Dabsyl**] was obtained at each timepoint i according to Equation 2:

$$[\boldsymbol{CORM\text{–}Dabsyl}] = \frac{A_i^{487} - A_\infty^{487}}{l * (\varepsilon_{CORM-Dabsyl}^{487} - \varepsilon_{product}^{487})} \; (Equation\ 2)$$

where A_i^{487} is the absorbance at 487 nm at each timepoint i or at the end of the reaction (∞) and ε^{487} is the extinction coefficient at 487 nm (3.4×10^4 and 1.3×10^4 for **CORM-Dabsyl** and the photoproduct, respectively). φ had a value of 0.5% ± 0.1% under 424 nm irradation. Since the photoreaction is accompanied by the release of three carbonyls (see main text), the quantum yield of CO-release was 1.5 ± 0.2% under 424 nm irradation.

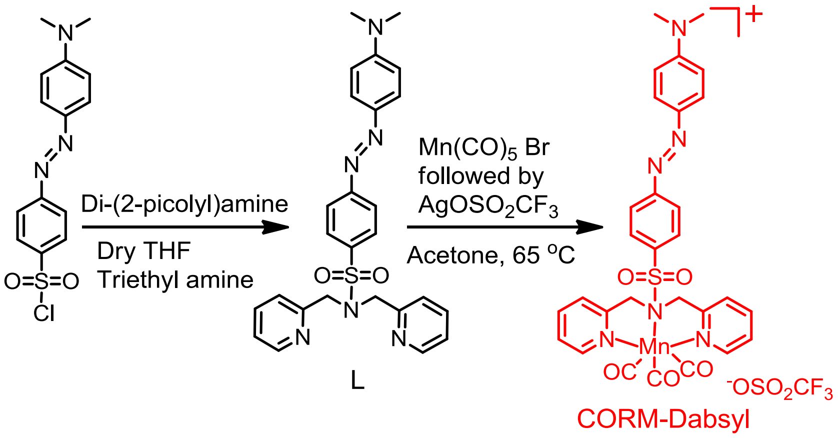

Scheme 1. Synthesis of **L** and **CORM-Dabsyl**.

^{1}H NMR spectrum of L

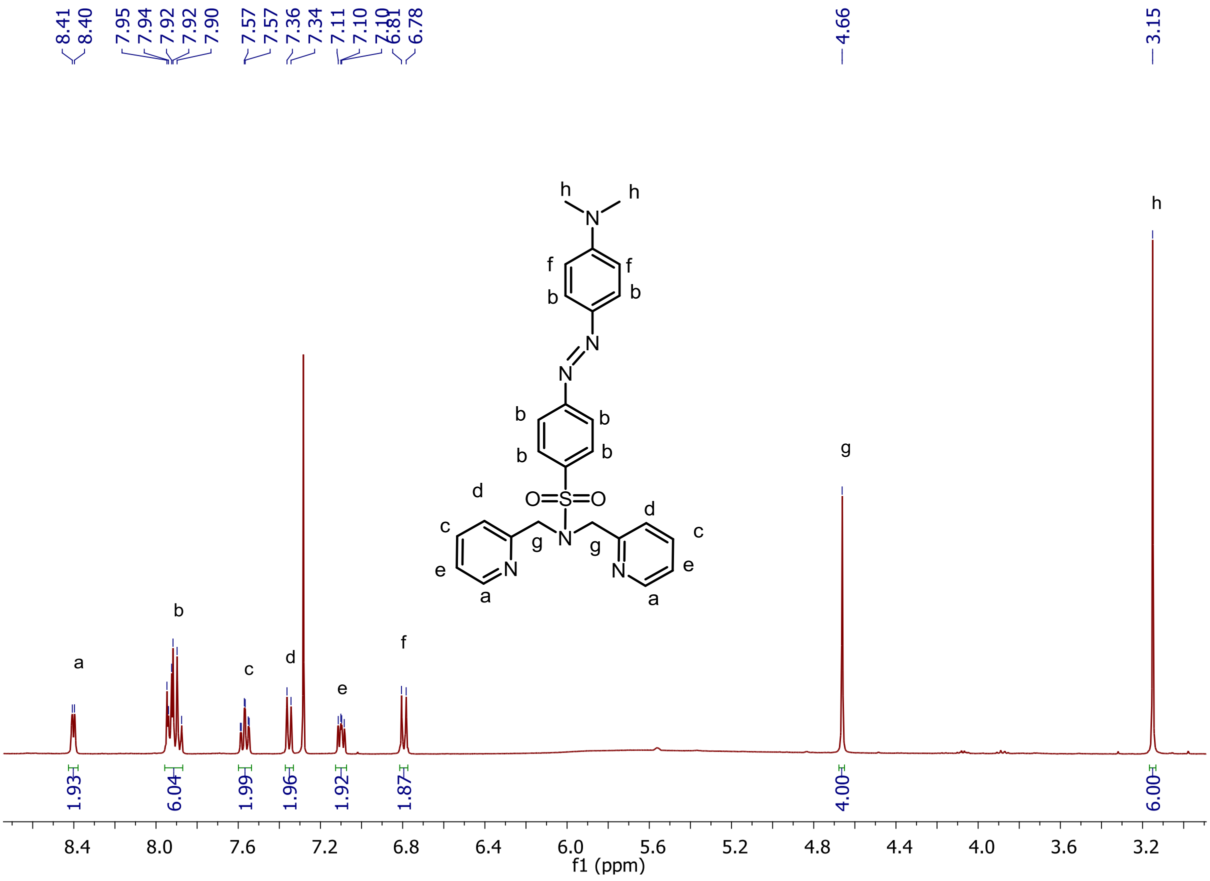

Figure S 1. ^{1}H NMR spectrum of **L** in $CDCl_3$.

^{13}C NMR spectrum of L

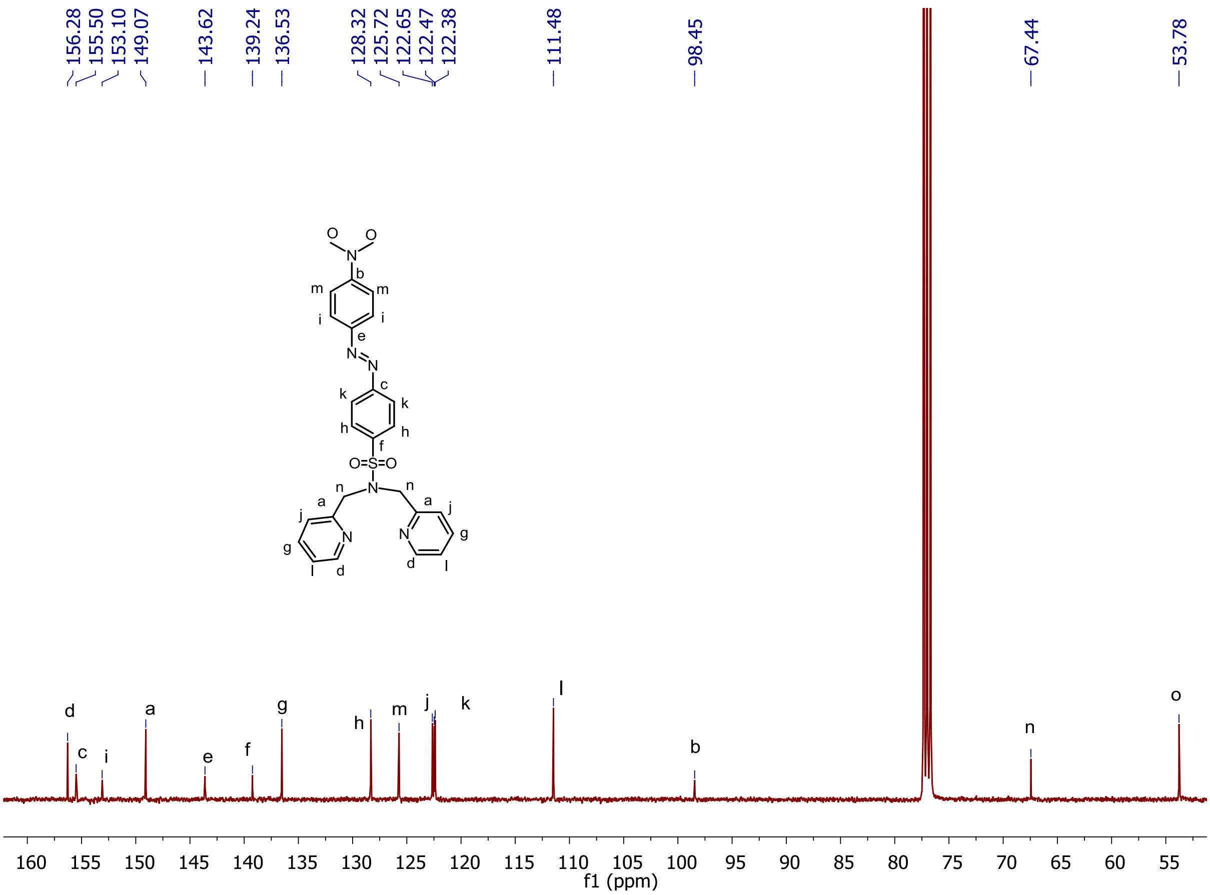

Figure S 2. ^{13}C NMR spectrum of **L** in $CDCl_3$.

High resolution mass spectrum of L

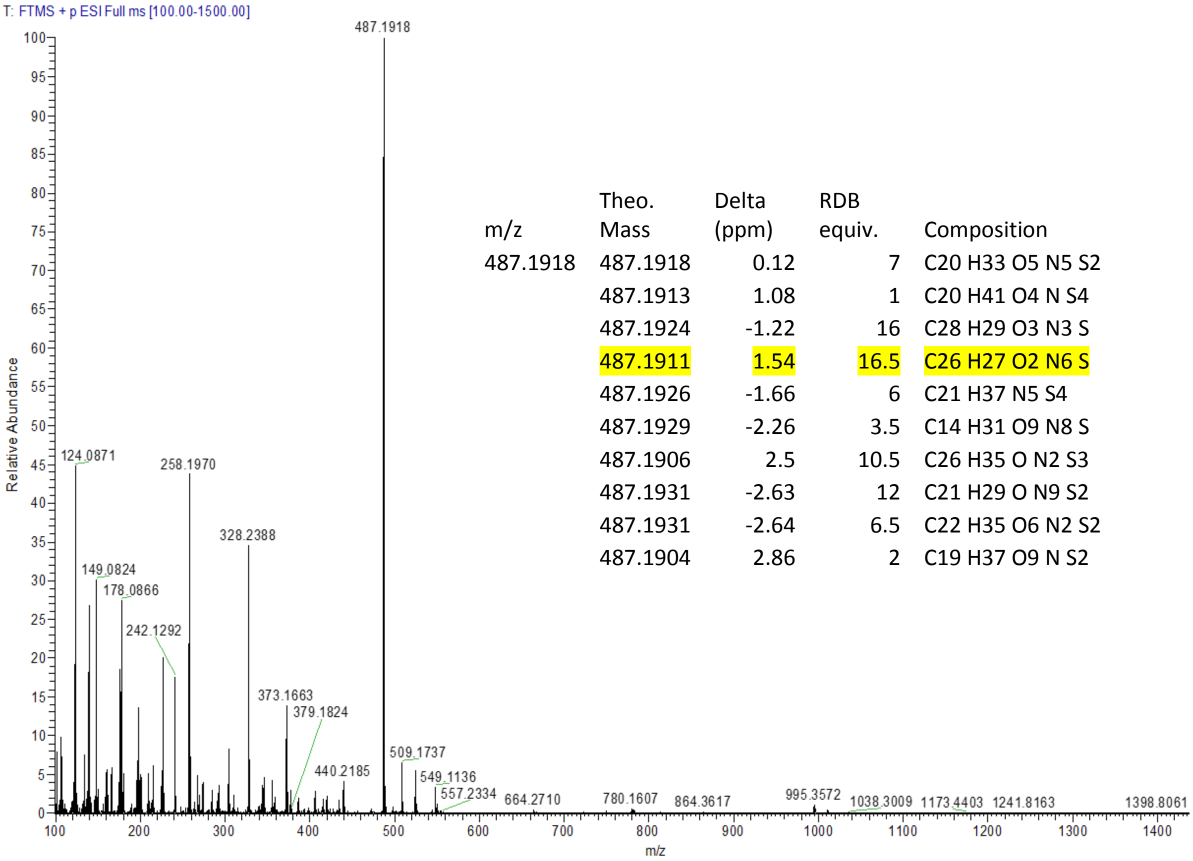

m/z	Theo. Mass	Delta (ppm)	RDB equiv.	Composition
487.1918	487.1918	0.12	7	C20 H33 O5 N5 S2
	487.1913	1.08	1	C20 H41 O4 N S4
	487.1924	-1.22	16	C28 H29 O3 N3 S
	487.1911	1.54	16.5	C26 H27 O2 N6 S
	487.1926	-1.66	6	C21 H37 N5 S4
	487.1929	-2.26	3.5	C14 H31 O9 N8 S
	487.1906	2.5	10.5	C26 H35 O N2 S3
	487.1931	-2.63	12	C21 H29 O N9 S2
	487.1931	-2.64	6.5	C22 H35 O6 N2 S2
	487.1904	2.86	2	C19 H37 O9 N S2

Figure S 3. High resolution mass spectrum of **L** in CH_3CN.

IR spectrum of L

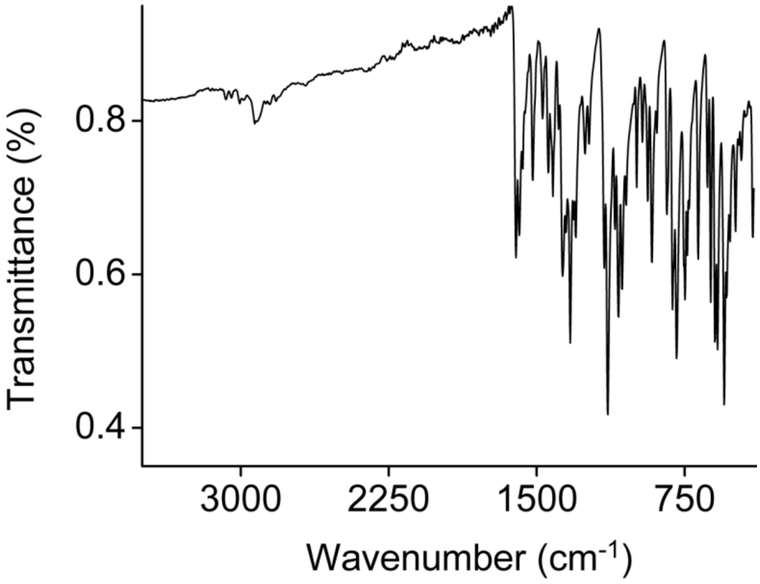

Figure S 4. IR spectrum of **L**.

^{1}H NMR spectrum of CORM-Dabsyl

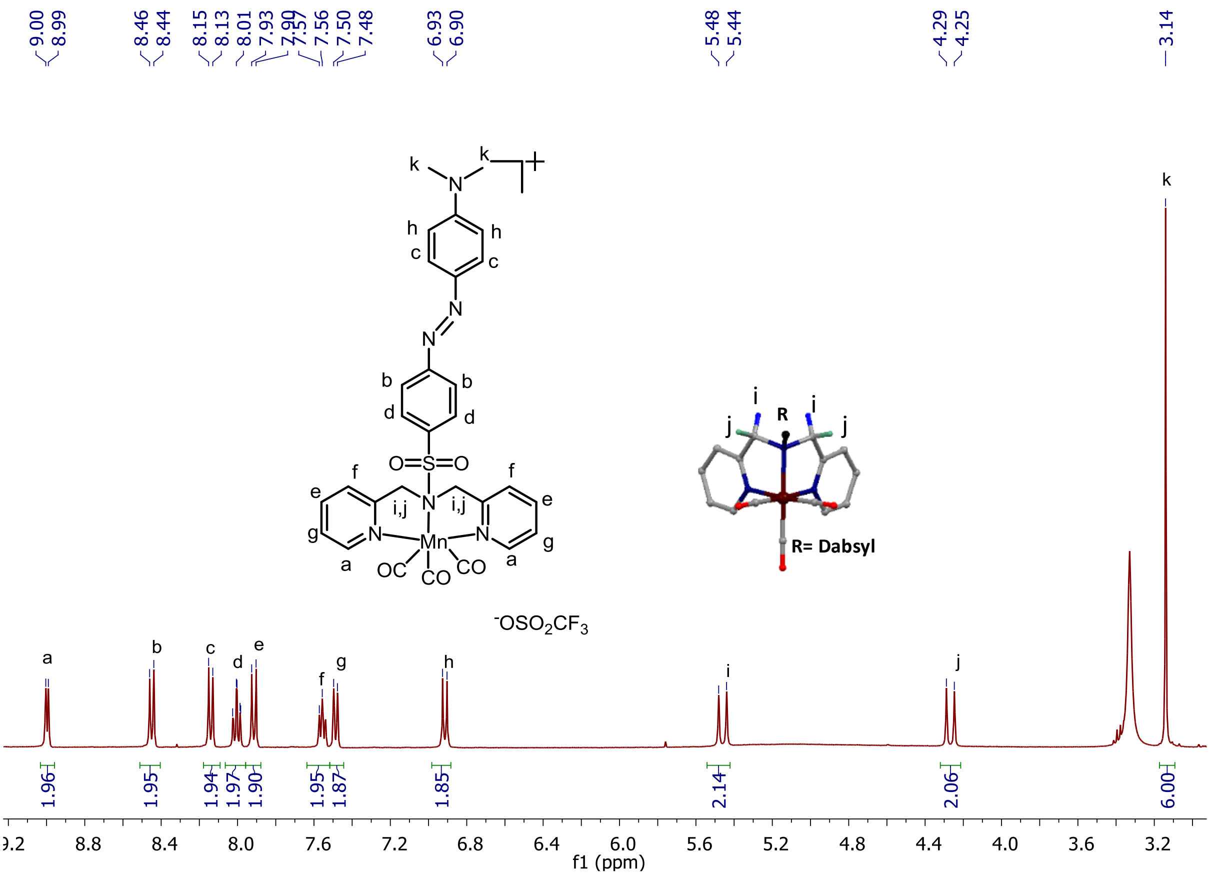

Figure S 5. ^{1}H NMR spectrum of **CORM-Dabsyl** in DMSO-d_6.

^{13}C NMR spectrum of CORM-Dabsyl

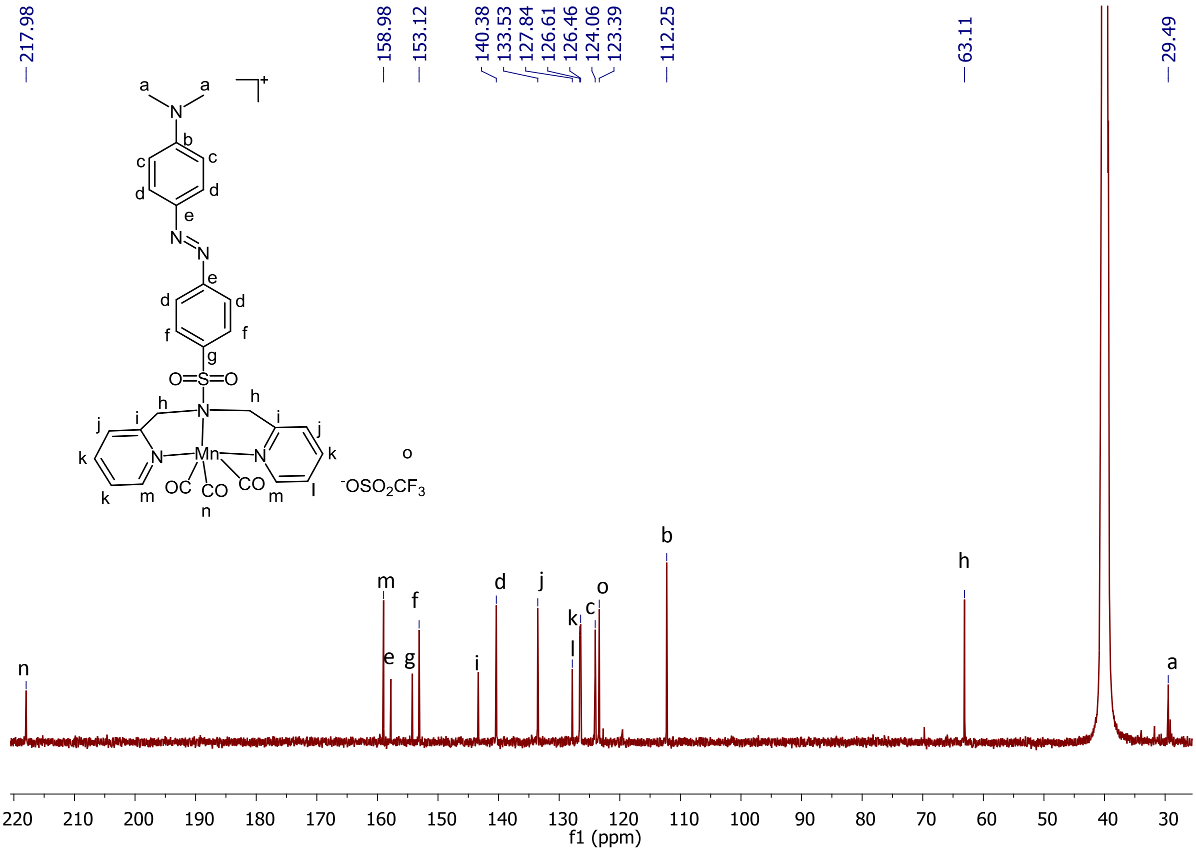

Figure S 6. ^{13}C NMR spectrum of **CORM-Dabsyl** in DMSO-d_6.

HRMS spectrum of CORM-Dabsyl

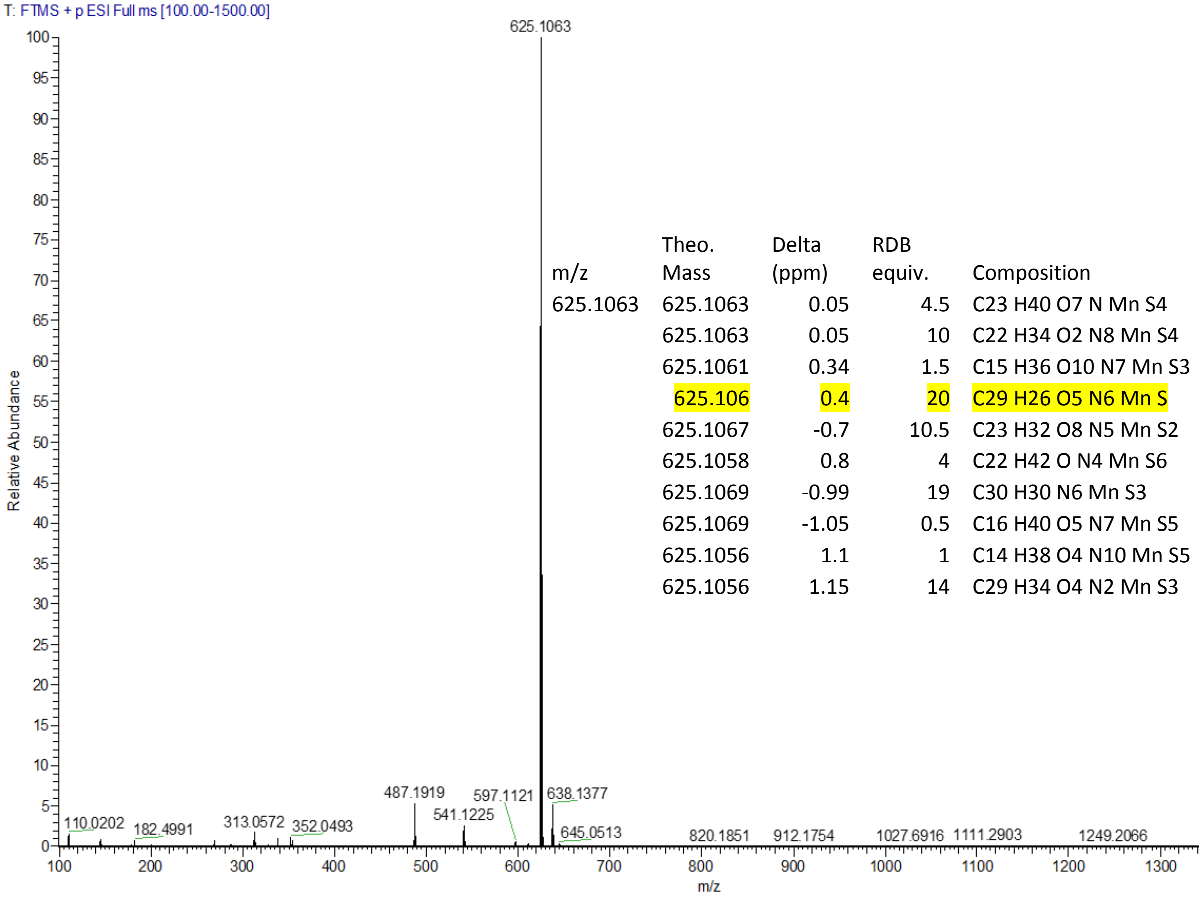

m/z	Theo. Mass	Delta (ppm)	RDB equiv.	Composition
625.1063	625.1063	0.05	4.5	C23 H40 O7 N Mn S4
	625.1063	0.05	10	C22 H34 O2 N8 Mn S4
	625.1061	0.34	1.5	C15 H36 O10 N7 Mn S3
	625.106	0.4	20	C29 H26 O5 N6 Mn S
	625.1067	-0.7	10.5	C23 H32 O8 N5 Mn S2
	625.1058	0.8	4	C22 H42 O N4 Mn S6
	625.1069	-0.99	19	C30 H30 N6 Mn S3
	625.1069	-1.05	0.5	C16 H40 O5 N7 Mn S5
	625.1056	1.1	1	C14 H38 O4 N10 Mn S5
	625.1056	1.15	14	C29 H34 O4 N2 Mn S3

Figure S 7. HRMS spectrum of **CORM-Dabsyl** in CH_3CN. Inset chemical composition

^{19}F NMR spectrum of CORM-Dabsyl

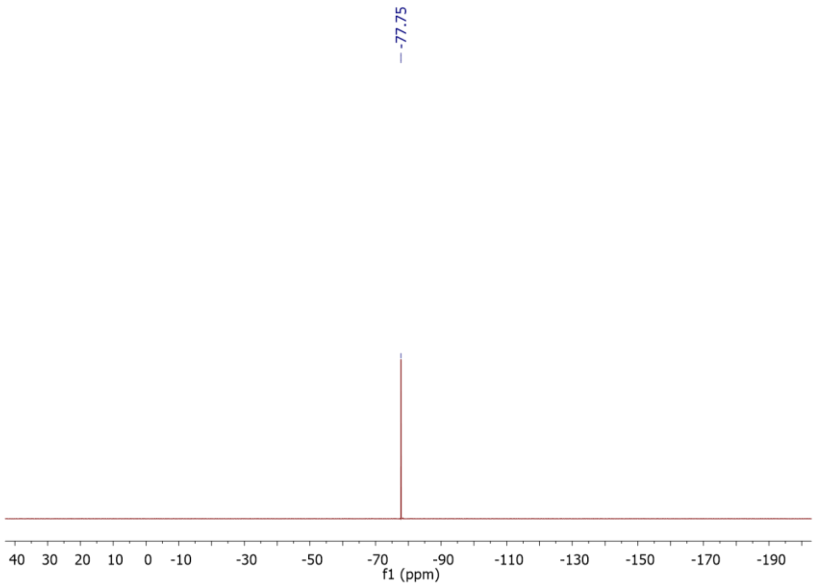

Figure S 8 .^{19}F NMR spectrum of **CORM-Dabsyl** in DMSO-d_6.

IR spectrum of CORM-Dabsyl

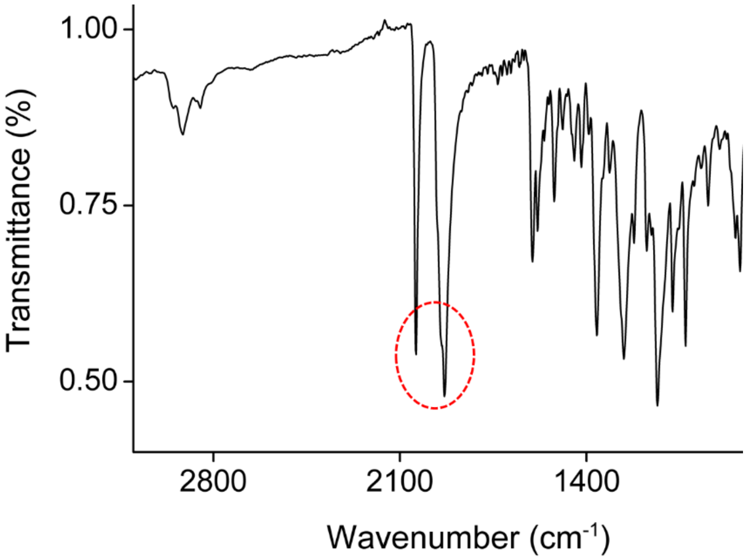

Figure S 9. IR spectrum of **CORM-Dabsyl**.

UV-Vis spectral changes of CORM-Dabsyl at photolysis as well as dark conditions

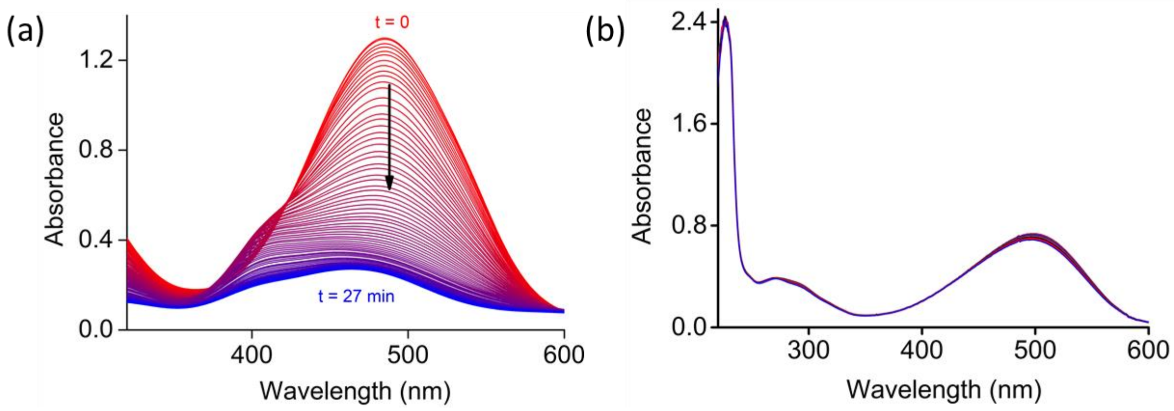

Figure S 10. (a) Electronic absorption spectral traces of **CORM-Dabsyl** (50 μM) in aq.-PB: DMSO (99:1, *v*/*v*) during blue light irradiation (424 nm, 23 mW). Time between each spectra is 40 s. (b) Electronic absorption spectral traces of **CORM-Dabsyl** (15 μM) in nitrogen bubbled aq.-PB: DMSO (99:1, *v*/*v*) recorded at dark conditions for every 10 min intervals.

UV-Vis Spectral changes of L upon illumination at 405 nm

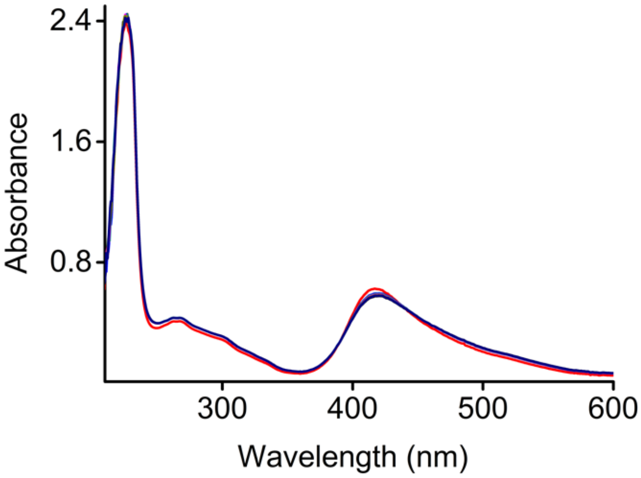

Figure S 11. Electronic absorption spectral traces of **L** (15 μM) in aq.-PB: DMSO (99:1, *v*/*v*, pH 7.4) upon illumination with 405 nm (8 mW cm^{-2}) at 20 sec intervals.

Influence of sodium dithionite for CORM-Dabsyl on CO-releasing

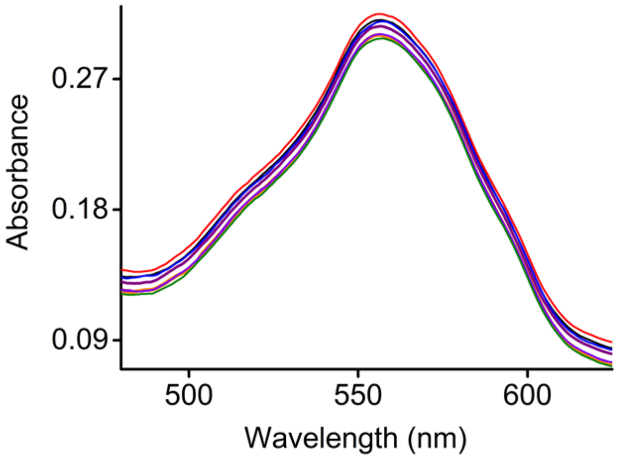

Figure S 12. Electronic absorption spectral traces of **CORM-Dabsyl** (15 μM) in presence of sodium dithionite and myoglobin (60 μM) recorded at dark conditions for every 10 min intervals in nitrogen bubbled aq.-PB: DMSO (99:1, *v*/*v*, pH 7.4).

UV-Vis spectral changes of CORM-Dabsyl at different irradiation wavelengths

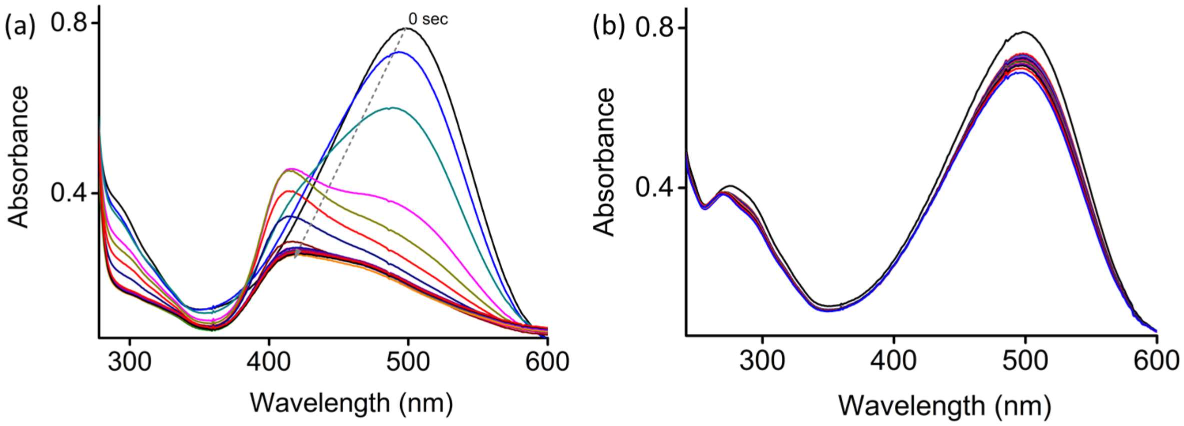

Figure S 13. Time-dependent UV-Vis spectra of **CORM-Dabsyl** (15 µM) under different irradiation sources at (a) 365 nm and (b) 480 nm maintaining equal light intensity (8 mW cm^{-2}) recorded in aq.-PB: DMSO (99:1, *v*/*v*, pH 7.4). Each spectrum was recorded for every 20 sec intervals.

ESR spectrum of CORM-Dabsyl before and after irradiation

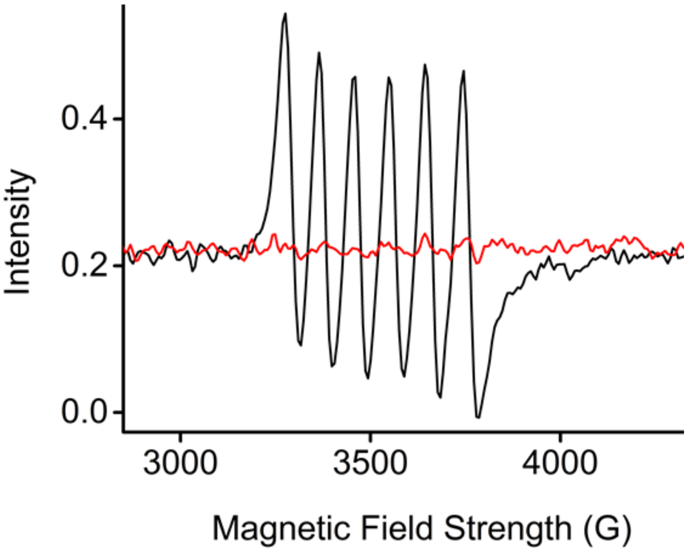

Figure S 14. X-band ESR spectra (at 295 K) of **CORM-Dabsyl** (4 mM) before (red line, **CORM-Dabsyl** exist in Mn^{I}-d^{6}, low spin, diamagnetic) and after photolysis (black line, **CORM-Dabsyl** exist in Mn^{II}-d^{5}, high spin, paramagnetic) in DMSO: water (1:1, *v/v*). Microwave frequency, 9.87 GHz; modulation amplitude, 0.6 Mt; modulation frequency, 100 kHz and power of the microwave source, 1,262 mW.

^{1}H NMR and ^{13}C NMR spectral changes of CORM-Dabsyl before and after irradiation

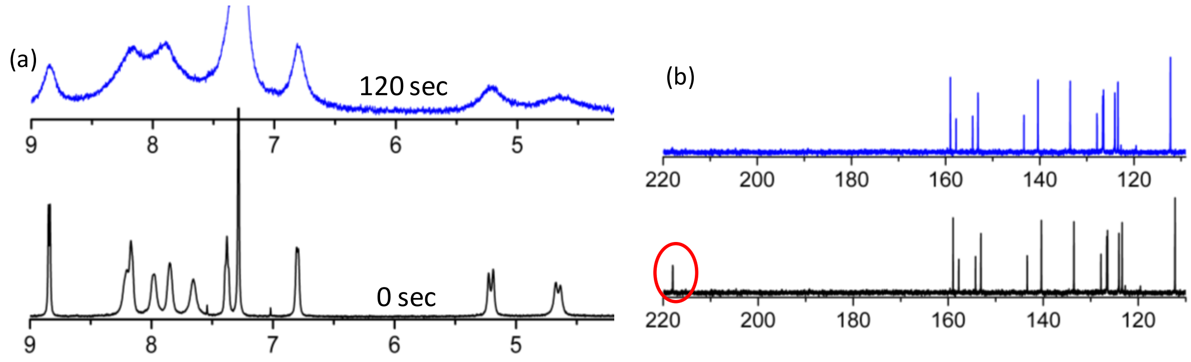

Figure S 15. (a) Time-dependent partial ^{1}H NMR spectra of **CORM-Dabsyl** before (black) and after (blue) photolysis (405 nm, 8 mW cm^{-2}). (b) ^{13}C NMR spectra of **CORM-Dabsyl** before (black) and after (blue) photolysis (405 nm, 8 mW cm^{-2}).

IR spectra of CORM-Dabsyl before and after irradiation

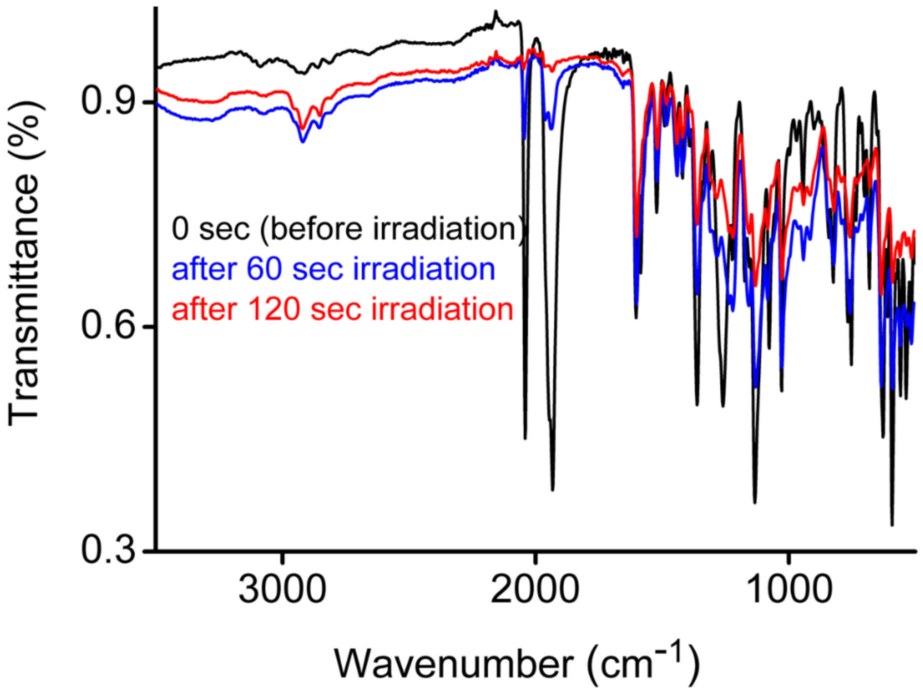

Figure S 16. ATR-IR spectra of **CORM-Dabsyl** before (black line) and after irradiation (blue and red line). The spectra show the disappearance of the CO vibrational bands (between 1900–2100 cm^{-1}) after the irradiation at 405 nm.

HRMS spectrum of CORM-Dabsyl after irradiation

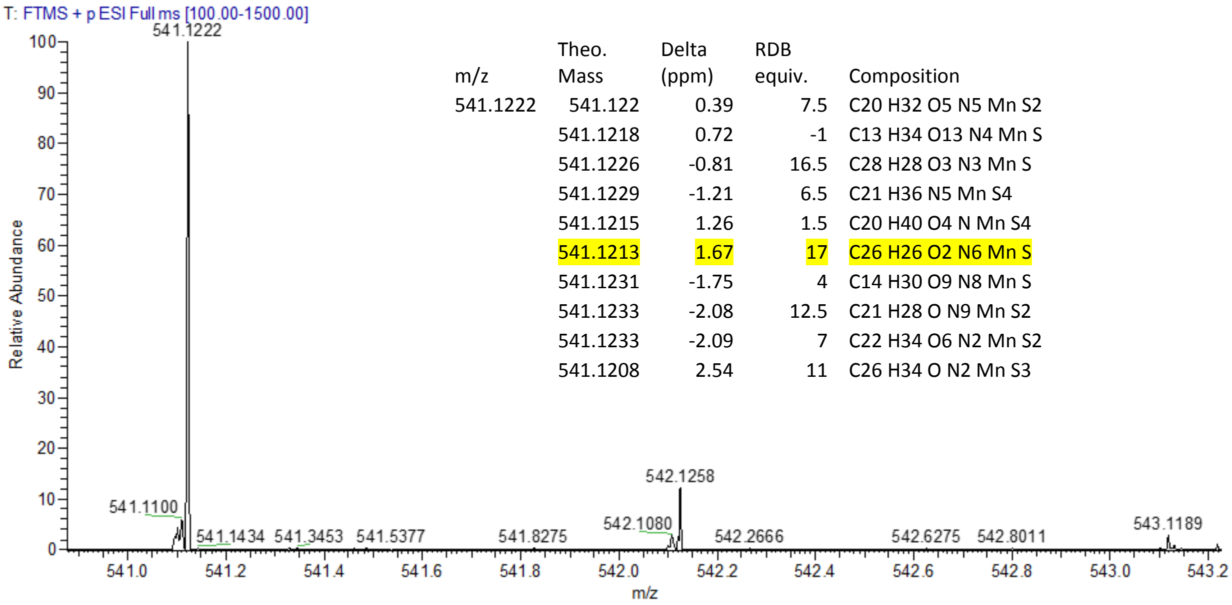

m/z	Theo. Mass	Delta (ppm)	RDB equiv.	Composition
541.1222	541.122	0.39	7.5	C20 H32 O5 N5 Mn S2
	541.1218	0.72	-1	C13 H34 O13 N4 Mn S
	541.1226	-0.81	16.5	C28 H28 O3 N3 Mn S
	541.1229	-1.21	6.5	C21 H36 N5 Mn S4
	541.1215	1.26	1.5	C20 H40 O4 N Mn S4
	541.1213	1.67	17	C26 H26 O2 N6 Mn S
	541.1231	-1.75	4	C14 H30 O9 N8 Mn S
	541.1233	-2.08	12.5	C21 H28 O N9 Mn S2
	541.1233	-2.09	7	C22 H34 O6 N2 Mn S2
	541.1208	2.54	11	C26 H34 O N2 Mn S3

Figure S 17. HRMS spectrum of **CORM-Dabsyl** in CH_3CN. Inset Chemical Composition.

Stability of paper strip and IR response of paper strip before and after irradation

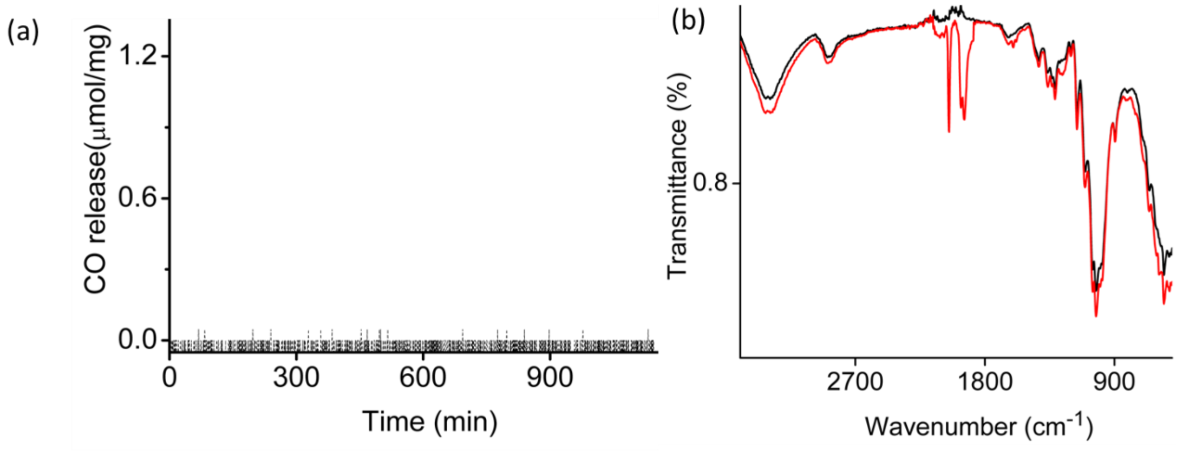

Figure S 18. (a) Amount of CO released as a function of time of a paper strip loaded with **CORM-Dabsyl** in ambient light conditions without LED irradiation in a closed desiccator. (b) IR response of paper strip before (red line) and after (black line) irradiation.

Leaching experiment with CORM-Dabsyl loaded paper strip

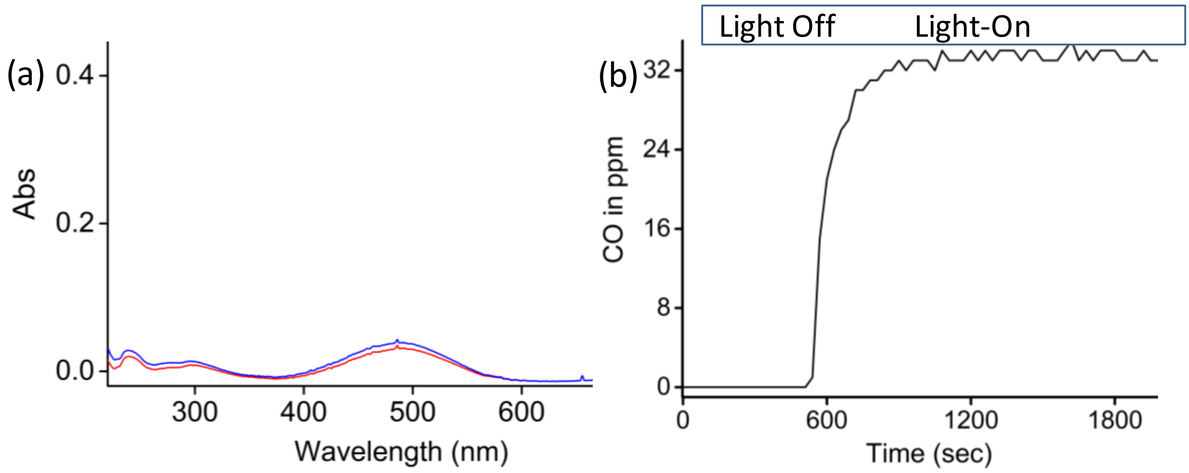

Figure S 19. (a) UV/Vis measurement of the supernatant of an aqueous solution in which the paper strip has been immersed at 0 min (red) and after 15 min (blue); (b) Amount of CO released (in ppm) from immersed paper strip (paper strip loaded with **CORM-Dabsyl** (1.0 mg) soaked in water for 15 min and allowed it for air dry) under LED irradiation in a closed desiccator.

Theoretical results

Computational Details

Coligand L.

State	λ / nm	f	Transition	Weight / %
S_1	521	0.0003	n_{azo} (127) → π^*_{dab} (129)	97
S_2	424	1.2985	π_{dab} (128) → π^*_{dab} (129)	98
S_3	310	0.0192	π_{py} (126) → π^*_{dab} (129)	56
			$n_{N,py}$ (125) → π^*_{dab} (129)	22
			$n_{N,py}$ (124) → π^*_{dab} (129)	15
S_4	309	0.0219	π_{py} (126) → π^*_{dab} (129)	40
			$n_{N,py}$ (125) → π^*_{dab} (129)	24
			$n_{N,py}$ (124) → π^*_{dab} (129)	19
S_5	306	0.0021	π_{dab} (120) → π^*_{dab} (129)	49
			π_{dab} (121) → π^*_{dab} (129)	17
			π_{dab} (128) → π^*_{dab} (133)	15
S_6	303	0.0135	π_{dab} (121) → π^*_{dab} (129)	53
			π_{dab} (120) → π^*_{dab} (129)	13

Table 1. Calculated excited singlet states above 300 nm, wave lengths (λ / nm), oscillator strengths (f), main transitions and weights in the Franck-Condon region of **L**.

CORM-Dabsyl.

State	λ / nm	*f*	Transition	Weight / %
S_1	548	0.0005	n_{Azo} (160) → π^*_{dab} (162)	95
S_2	464	1.3538	π_{dab} (161) → π^*_{dab} (162)	98
S_3	457	0.0148	π_{dab} (161) → π^*_{py} (163)	99
S_4	418	0.0003	d_π (157) → π^*_{CO} / d_{z2} (167)<br>d_π (159) → π^*_{CO} / d_{x2-y2} (168)	45<br>18
S_5	410	0.0056	d_π (158) → π^*_{CO} / d_{z2} (167)<br>d_π (159) → π^*_{CO} / d_{z2} (167)	39<br>19
S_6	396	0.0034	d_π (159) → π^*_{CO} / d_{x2-y2} (168)<br>d_π (157) → π^*_{CO} / d_{z2} (167)	45<br>10
S_7	392	0.0000	π_{dab} (161) → π^*_{py} (164)	100
S_8	387	0.0061	π_{dab} (161) → π^*_{py} (165)	99
S_9	384	0.0172	d_π (159) → π^*_{CO} / d_{z2} (167)<br>d_π (159) → π^*_{py} (163)<br>d_π (158) → π^*_{CO} / d_{z2} (167)	35<br>26<br>19
S_{10}	357	0.0077	d_π (158) → π^*_{py} (163)<br>d_π (159) → π^*_{py} (163)<br>d_π (159) → π^*_{CO} / d_{z2} (167)	38<br>36<br>14

Table 2. Calculated excited singlet states above 300 nm, wave lengths (λ / nm), oscillator strengths (f), main transitions and weights in the Franck-Condon region of **CORM-Dabsyl**.

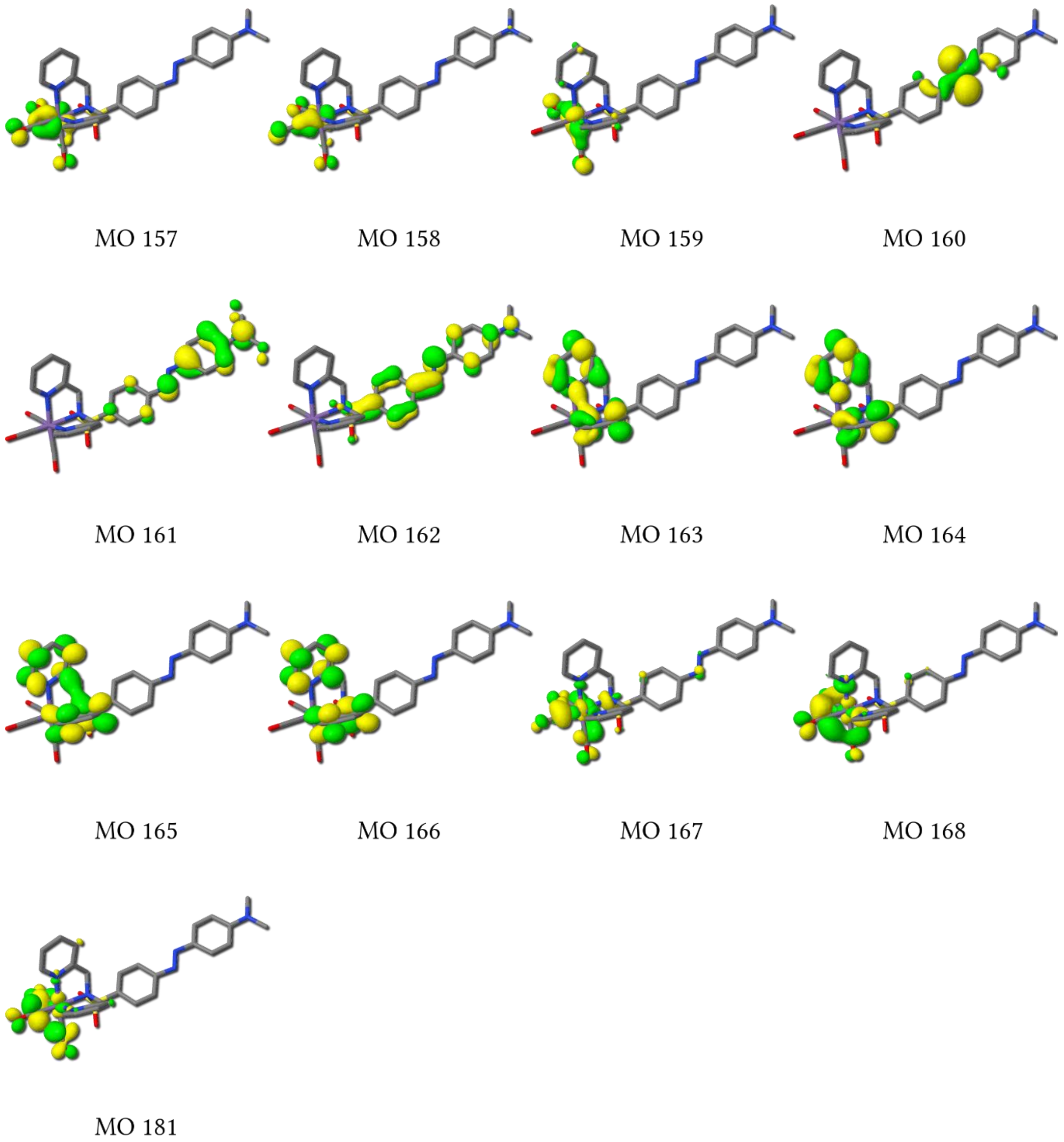

Figure S 20. Selected molecular orbitals (ρ = 0.04) of **CORM-Dabsyl**. Hydrogen atoms were omitted for clarity. Color code: C = grey, N = blue, O = red, S = yellow, Mn = purple. The d_π-orbitals (MOs 157 – 159) are responsible for the π-back bonding between the Mn and the CO-ligands. Excitations from the d_π-orbitals into MOs 167 and 168 lead to photodissociation of the CO-ligands.

iCORM-Dabsyl.

State	ΔE / eV	Configuration	%
D_0	+1.79	$(n_{Azo})^2(\pi_1)^2(\pi_2)^2(\pi_4)^2(\pi_3)^2(d_1)^0(d_2)^u(d_3)^2(d_4)^2(d_5)^0(\pi_3^*)^0(\pi_2^*)^0(\pi_1^*)^0(\pi_4^*)^0$	81
D_1	+1.94	$(n_{Azo})^2(\pi_1)^2(\pi_2)^2(\pi_4)^2(\pi_3)^2(d_1)^0(d_2)^2(d_3)^u(d_4)^2(d_5)^0(\pi_3^*)^0(\pi_2^*)^0(\pi_1^*)^0(\pi_4^*)^0$	79
D_2	+2.02	$(n_{Azo})^2(\pi_1)^2(\pi_2)^2(\pi_4)^2(\pi_3)^2(d_1)^0(d_2)^2(d_3)^2(d_4)^u(d_5)^0(\pi_3^*)^0(\pi_2^*)^0(\pi_1^*)^0(\pi_4^*)^0$	80
Q_0	+2.48	$(n_{Azo})^2(\pi_1)^2(\pi_3)^2(\pi_2)^2(\pi_4)^2(d_1)^u(d_2)^u(d_3)^2(d_4)^u(d_5)^0(\pi_4^*)^0(\pi_2^*)^0(\pi_1^*)^0(\pi_3^*)^0$	82
Q_1	+2.56	$(n_{Azo})^2(\pi_1)^2(\pi_3)^2(\pi_2)^2(\pi_4)^2(d_1)^u(d_2)^u(d_3)^u(d_4)^2(d_5)^0(\pi_4^*)^0(\pi_2^*)^0(\pi_1^*)^0(\pi_3^*)^0$	61
		$(n_{Azo})^2(\pi_1)^2(\pi_3)^2(\pi_2)^2(\pi_4)^u(d_1)^d(d_2)^u(d_3)^u(d_4)^u(d_5)^u(\pi_4^*)^d(\pi_2^*)^0(\pi_1^*)^0(\pi_3^*)^0$	12
Q_2	+2.63	$(n_{Azo})^2(\pi_1)^2(\pi_3)^2(\pi_2)^2(\pi_4)^2(d_1)^u(d_2)^u(d_3)^u(d_4)^2(d_5)^0(\pi_4^*)^0(\pi_2^*)^0(\pi_1^*)^0(\pi_3^*)^0$	19
		$(n_{Azo})^2(\pi_1)^2(\pi_3)^2(\pi_2)^2(\pi_4)^u(d_1)^d(d_2)^u(d_3)^u(d_4)^u(d_5)^u(\pi_4^*)^d(\pi_2^*)^0(\pi_1^*)^0(\pi_3^*)^0$	38
		$(n_{Azo})^2(\pi_1)^2(\pi_3)^2(\pi_2)^2(\pi_4)^u(d_1)^u(d_2)^d(d_3)^u(d_4)^u(d_5)^u(\pi_4^*)^d(\pi_2^*)^0(\pi_1^*)^0(\pi_3^*)^0$	13
$Sextet_0$	0.00	$(n_{Azo})^2(\pi_1)^2(\pi_2)^2(\pi_3)^2(\pi_4)^2(d_1)^u(d_2)^u(d_3)^u(d_4)^u(d_5)^u(\pi_4^*)^0(\pi_2^*)^0(\pi_1^*)^0(\pi_3^*)^0$	85
$Sextet_1$	+1.97	$(n_{Azo})^2(\pi_1)^2(\pi_2)^2(\pi_3)^2(\pi_4)^u(d_1)^d(d_2)^u(d_3)^u(d_4)^u(d_5)^u(\pi_4^*)^u(\pi_2^*)^0(\pi_1^*)^0(\pi_3^*)^0$	26
		$(n_{Azo})^2(\pi_1)^2(\pi_2)^2(\pi_3)^2(\pi_4)^u(d_1)^u(d_2)^u(d_3)^u(d_4)^u(d_5)^u(\pi_4^*)^d(\pi_2^*)^0(\pi_1^*)^0(\pi_3^*)^0$	31
$Sextet_2$	+2.14	$(n_{Azo})^u(\pi_1)^2(\pi_2)^2(\pi_3)^2(\pi_4)^2(d_1)^d(d_2)^u(d_3)^u(d_4)^u(d_5)^u(\pi_4^*)^u(\pi_2^*)^0(\pi_1^*)^0(\pi_3^*)^0$	31
		$(n_{Azo})^u(\pi_1)^2(\pi_2)^2(\pi_3)^2(\pi_4)^2(d_1)^u(d_2)^d(d_3)^u(d_4)^u(d_5)^u(\pi_4^*)^u(\pi_2^*)^0(\pi_1^*)^0(\pi_3^*)^0$	11
		$(n_{Azo})^u(\pi_1)^2(\pi_2)^2(\pi_3)^2(\pi_4)^2(d_1)^u(d_2)^u(d_3)^u(d_4)^u(d_5)^u(\pi_4^*)^d(\pi_2^*)^0(\pi_1^*)^0(\pi_3^*)^0$	38

Table 3. Energy differences (ΔE / eV) and configurations for the first three doublet (*D*), quartet (*Q*) and sextet (*Sextet*) states of **iCORM-Dabysl** obtained at MS-RASPT2 level using a RAS (15,2,2;4,7,3) partitioning of the active space (Figure S 20-22). All energies are given in respect to the total energy of the sextet ground state ($Sextet_0$). Superscripts denote the electronic occupation of the respective orbital in the active space for a given configuration (2 = doubly occupied, 0 = empty, u = up spin, d = down spin). The $_{dab}$-subscript for the π and π*-orbitals located on the coligand was omitted for clarity.

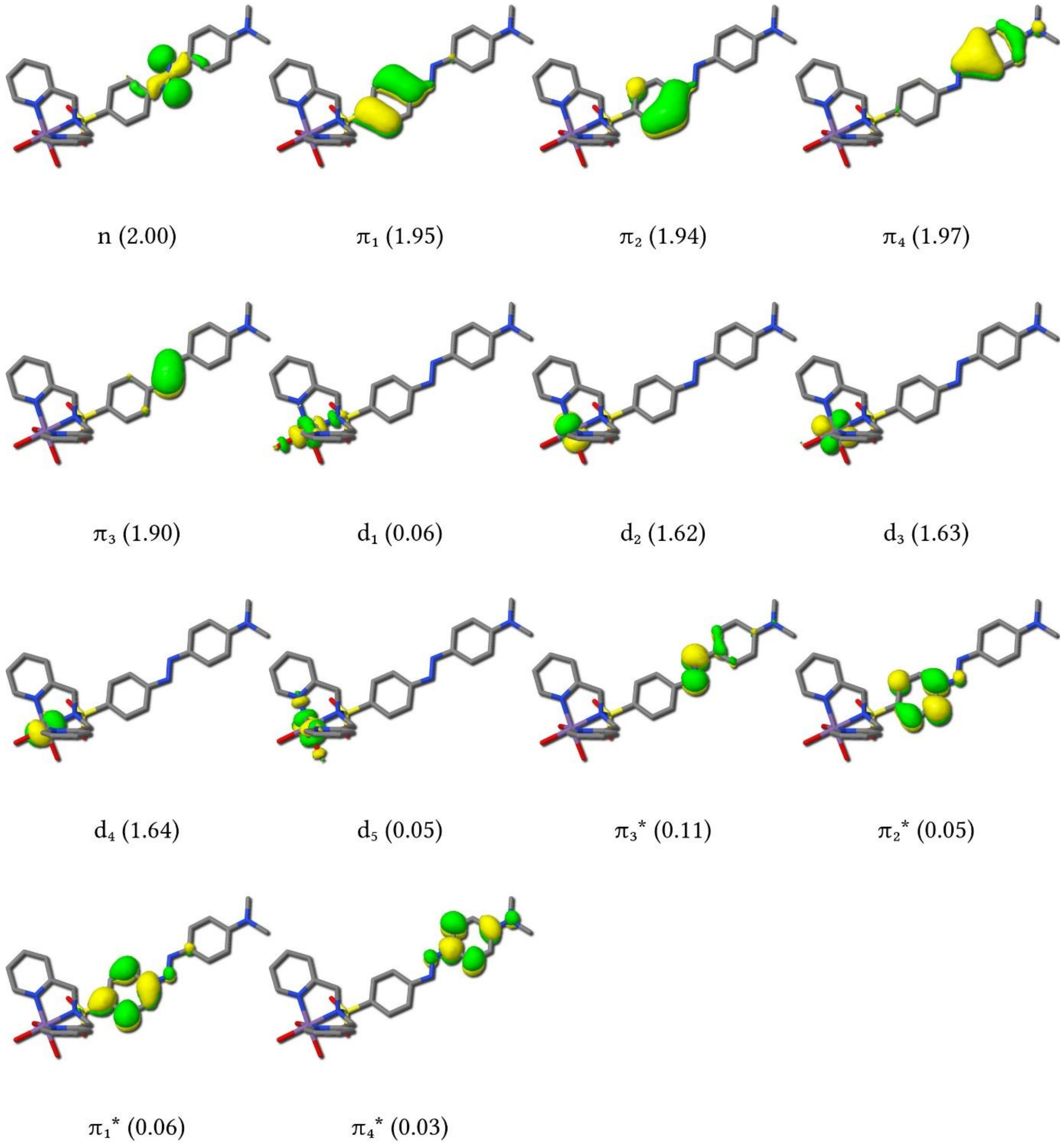

Figure S 21. Illustration of the RAS (15,2,2;4,7,3) and average occupation numbers for the first three doublet states of **iCORM-Dabsyl**.

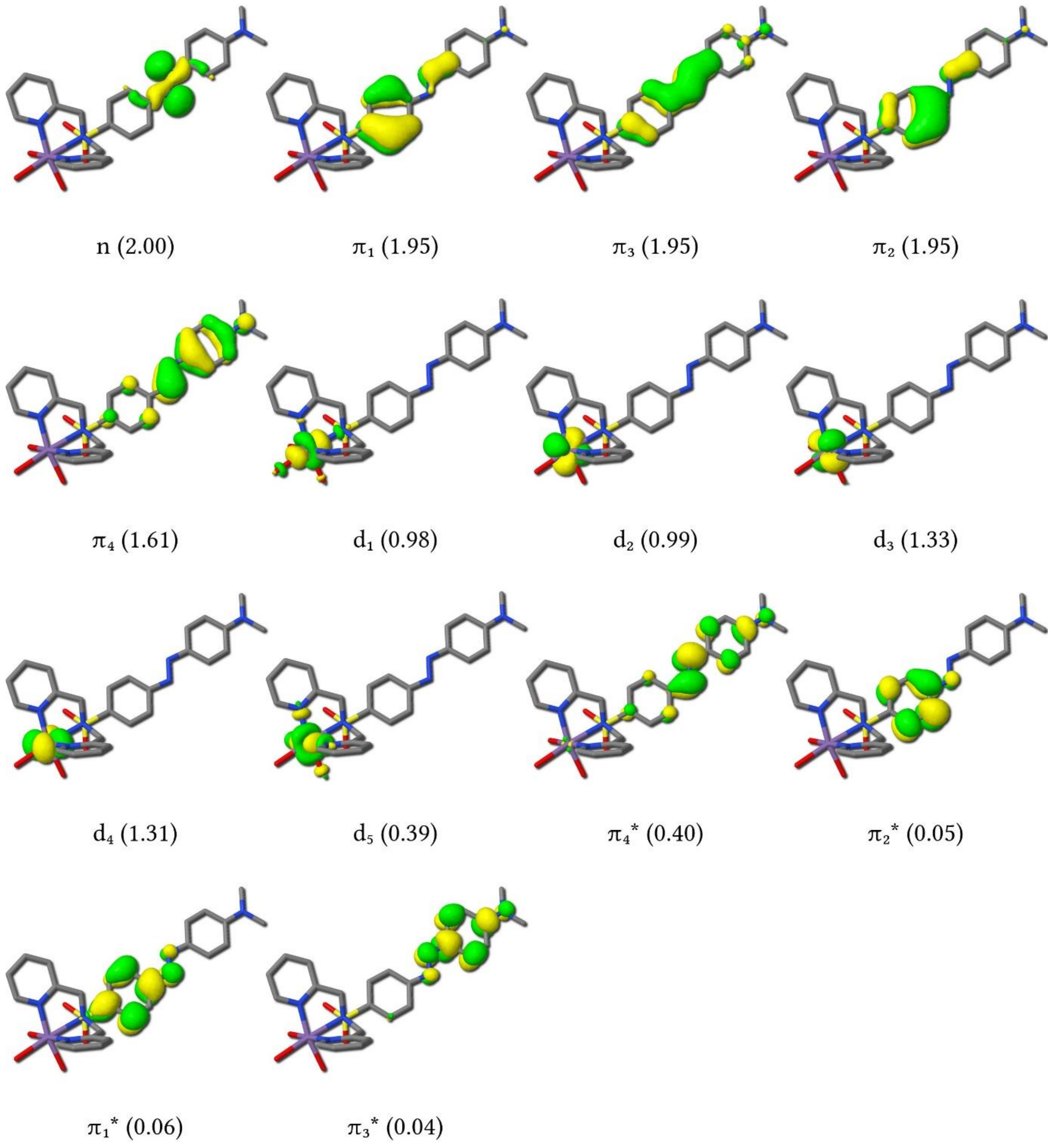

Figure S 22. Illustration of the RAS (15,2,2;4,7,3) and average occupation numbers for the first three quartet states of **iCORM-Dabsyl**.

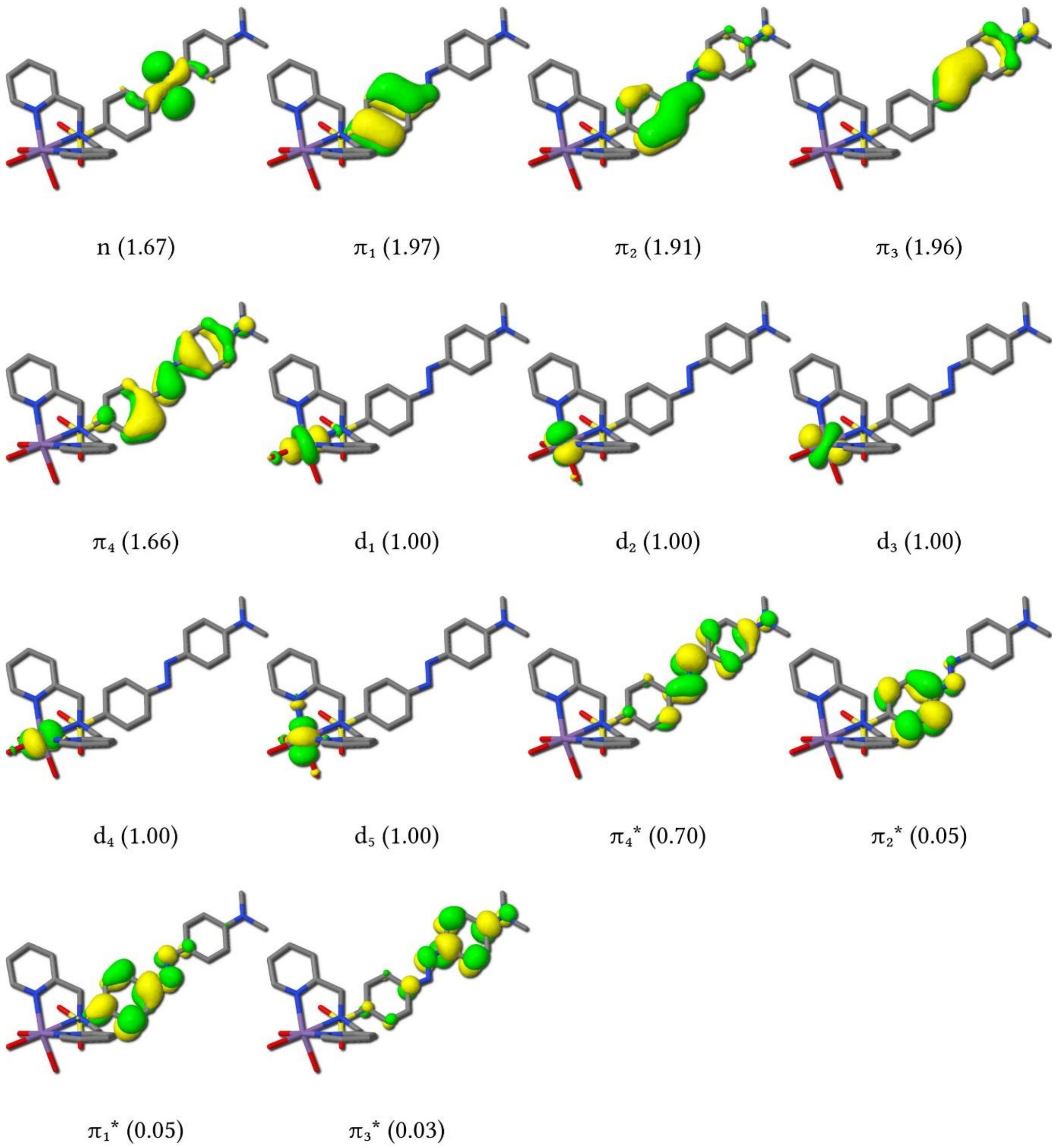

Figure S 23. Illustration of the RAS (15,2,2;4,7,3) and average occupation numbers for the first three sextet states of **iCORM-Dabsyl**.

Supporting information available: Crystallographic data deposited at the Cambridge Crystallographic Data Centre under CCDC-1534335 for **CORM-Dabsyl** contain the supplementary crystallographic data excluding structure factors; this data can be obtained free of charge via www.ccdc.cam.ac.uk/conts/retrieving.html (or from the Cambridge Crystallographic Data Centre, 12, Union Road, Cambridge CB2 1EZ, UK; fax: (+44) 1223-336-033; or deposit@ccdc.cam.ac.uk).

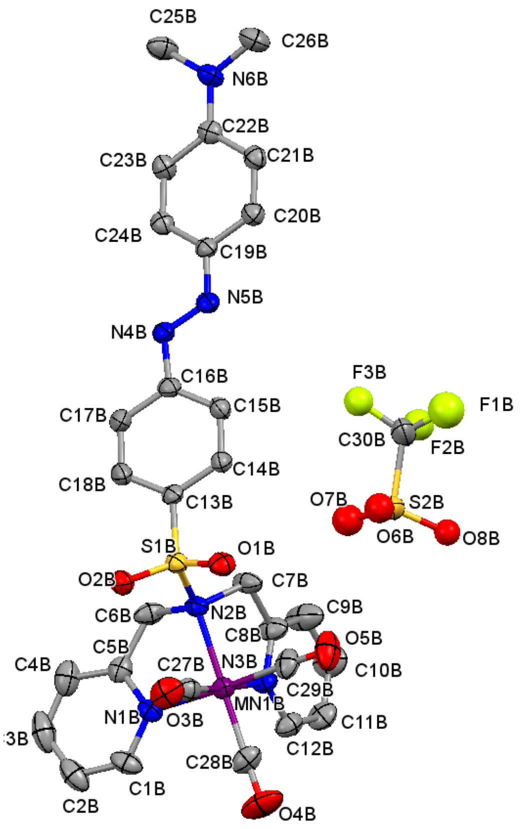

Figure S 24. Molecular structure and numbering scheme of **CORM-Dabsyl**.

References

1 (a) U. Reddy G, J. Axthelm, P. Hoffmann, N. Taye, S. Gläser, H. Görls, S. L. Hopkins, W. Plass, U. Neugebauer, S. Bonnet and A. Schiller, *J. Am. Chem. Soc.*, 2017, **139**, 4991; (b) S. Gläser, R. Mede, H. Görls, S. Seupel, C. Bohlender, R. Wyrwa, S. Schirmer, S. Dochow, G. U. Reddy, J. Popp, M. Westerhausen and A. Schiller, *Dalton Trans*., 2016, **45**,13222; (c) C. Bischof, T. Joshi, A. Dimri, L. Spiccia and U. Schatzschneider, *Inorg. Chem*. 2013, **52**, 9297.

2 (a) COLLECT, Data Collection Software; B.V. Nonius, Netherlands, 1998; (b) SADABS 2.10, Bruker-AXS inc., 2002, Madison, WI, U.S.A.

3 G. M. Sheldrick, *Acta Cryst.*, 2015, **C71**, 3-8.

4 A. L. Spek, *Acta Cryst.*, 2009, **D65**, 148-155.

5 Furche, R. Ahlrichs, C. Hättig, W. Klopper, M. Sierka and F. Weigend, *Wiley Interdisciplinary Reviews: Comput. Mol. Sci.*, 2014, **4**, 91.

6 C. F. Macrae, P. R. Edgington, P. McCabe, E. Pidcock, G. P. Shields, R. Taylor, M. Towler and J. V. de Streek, J. Appl. Cryst. 2006, 39, 453.

7 J. Tao, J. P. Perdew, V. N. Staroverov and G. E. Scuseria, *Phys. Rev. Lett*, 2003, **91**, 146401.

8 A. Schäfer, A. Klamt, D. Sattel, J. C. W. Lohrenz and F. Eckert, *Phys. Chem. Chem.Phys.*, 1970, **2**, 2187.

9 F. Weigend and R. Ahlrichs, *Phys. Chem. Chem. Phys.*, 2005, **7**, 3297.

10 (a) R. Ahlrichs, *Phys. Chem. Chem. Phys.*, 2004, **6**, 5119; (b) M. Sierka, A. Hogekamp and R. Ahlrichs, *J. Chem. Phys*, 2003, **118**, 9136.

11 S. Grimme, S. Ehrlich and L. Goerigk, *J Comput Chem*, 2011, **32**, 145.

12 Y. Zhao and D. G. Truhlar, *Theor Chem Acc*, 2007, **120**, 215.

13 P. A. Malmqvist, A. Rendell and B. O. Roos, *J. Phys. Chem.*, 1990, **94**, 5477.

14 P. A. Malmqvist, K. Pierloot, A. R. M. Shahi, C. J. Cramer and L. Gagliardi, *J. Chem. Phys.*, 2008, **128**, 204109.

15 F. Aquilante, J. Autschbach, R. K. Carlson, L. F. Chibotaru, M. G. Delcey, L. De Vico, I. Fdez. Galván, N. Ferré, L. M. Frutos, L. Gagliardi, M. Garavelli, A. Giussani, C. E. Hoyer, G. Li Manni, H. Lischka, D. Ma, P. Å. Malmqvist, T. Müller, A. Nenov, M. Olivucci, T. B. Pedersen, D. Peng, F. Plasser, B. Pritchard, M. Reiher, I. Rivalta, I. Schapiro, J. Segarra-Martí, M. Stenrup, D. G. Truhlar, L. Ungur, A. Valentini, S. Vancoillie, V. Veryazov, V. P. Vysotskiy, O. Weingart, F. Zapata and R. Lindh, *J. Comput. Chem.*, 2016, **37**, 506.

16 D. Peng and K. Hirao, *J. Chem. Phys.*, 2009, **130**, 044102.

17 (a) B. O. Roos, R. Lindh, P.-Å. Malmqvist, V. Veryazov and P.-O. Widmark, *J. Phys. Chem. A.*, 2004, **108**, 2851; (b) B. O. Roos, R. Lindh, P.-A. Malmqvist, V. Veryazov and P.-O. Widmark, *J. Phys. Chem. A.*, 2005, **109**, 6575.

18 F. Aquilante, R. Lindh and T. B. Pedersen, *J. Chem. Phys.*, 2007, **127**, 114107.

19 V. Sauri, L. Serrano-Andrés, A. R. M. Shahi, L. Gagliardi, S. Vancoillie and K. Pierloot, *J. Chem. Theory Compt.*, 2011, **7**, 153.

20 A. Bahreman, B. Limburg, M. A. Siegler, E. Bouwman and S. Bonnet, *Inorg. Chem*. 2013., **52**, 9456.
